# Detection and Molecular Characterization of Picobirnaviruses (PBVs) in the Mongoose: Identification of a Novel PBV Using an Alternative Genetic Code

**DOI:** 10.3390/v12010099

**Published:** 2020-01-15

**Authors:** Alyssa Kleymann, Anne A. M. J. Becker, Yashpal S. Malik, Nobumichi Kobayashi, Souvik Ghosh

**Affiliations:** 1 Department of Biomedical Sciences, Ross University School of Veterinary Medicine, P.O. Box 334 Basseterre, St. Kitts and Nevis, West Indies; AlyssaKleymann@students.rossu.edu (A.K.); abecker@rossvet.edu.kn (A.A.M.J.B.); 2 Division of Biological Standardization, Indian Veterinary Research Institute, Izatnagar, Bareilly, Uttar Pradesh 243122, India; malikyps@gmail.com; 3 Department of Hygiene, Sapporo Medical University, Sapporo, Hokkaido 060-8556, Japan; nkobayas@sapmed.ac.jp

**Keywords:** picobirnavirus, mongoose, gene segment-2, RNA-dependent RNA polymerase, genetic diversity, novel picobirnavirus-like sequence, alternative mitochondrial genetic code

## Abstract

We report high rates of detection (35.36%, 29/82) of genogroup-I (GI) picobirnaviruses (PBVs) in non-diarrheic fecal samples from the small Indian mongoose (*Urva auropunctata*). In addition, we identified a novel PBV-like RNA-dependent RNA polymerase (RdRp) gene sequence that uses an alternative mitochondrial genetic code (that of mold or invertebrate) for translation. The complete/nearly complete gene segment-2/RdRp gene sequences of seven mongoose PBV GI strains and the novel PBV-like strain were obtained by combining a modified non-specific primer-based amplification method with conventional RT-PCRs, facilitated by the inclusion of a new primer targeting the 3′-untranslated region (UTR) of PBV gene segment-2. The mongoose PBV and PBV-like strains retained the various features that are conserved in gene segment-2/RdRps of other PBVs. However, high genetic diversity was observed among the mongoose PBVs within and between host species. This is the first report on detection of PBVs in the mongoose. Molecular characterization of the PBV and PBV-like strains from a new animal species provided important insights into the various features and complex diversity of PBV gene segment-2/putative RdRps. The presence of the prokaryotic ribosomal binding site in the mongoose PBV genomes, and analysis of the novel PBV-like RdRp gene sequence that uses an alternative mitochondrial genetic code (especially that of mold) for translation corroborated recent speculations that PBVs may actually infect prokaryotic or fungal host cells.

## 1. Introduction

Picobirnaviruses (PBVs), members of the family *Picobirnaviridae*, are bi-segmented double-stranded RNA (dsRNA) viruses that have been reported in the feces/gut contents of a wide variety of host species, and rarely in the respiratory tract of cattle, humans, monkeys, and pigs [1,2,3,4,5,6]. Although the pathogenesis of PBVs have not yet been clearly elucidated, traditionally, PBVs have been considered as opportunistic enteric pathogens of mammals [1,5,6]. However, PBVs remain to be successfully propagated in mammalian cell cultures, or gnotobiotic animals [1,5,6]. On the other hand, PBVs have been detected in invertebrates, and recent studies have provided evidence that PBVs may in fact infect prokaryotic or fungal host cells [7,8,9,10,11].

The PBV genome consists of two gene segments [1,5,6]. The PBV gene segment-1 is ~2.2–2.7 kb in size and encodes the viral capsid protein and a putative polypeptide with unknown function, whilst the gene segment-2 is ~1.2–1.9 kb and encodes the viral RdRp. By using nucleotide (nt)/deduced amino acid (aa) sequence identities and phylogenetic analysis of gene segment-2, PBVs have been classified into at least two genogroups: genogroup-I (GI) and GII [5,6]. Genogroup-I PBV strains are more prevalent than GII PBVs [5,6].

The high genetic diversity of PBVs within and between host species has hampered conventional RT-PCR-based studies on whole-genome analysis of PBV strains [5,6]. Until recently, most studies on genetic diversity of PBVs were limited to analyses of short nt sequences (~201 bp of gene segment-2) which may not be sufficient to obtain conclusive information on the putative RdRps, or genetic make-up of PBVs [5,6,12]. However, by applying a non-specific primer-based amplification method, or next-generation sequencing, it has been possible to obtain the whole genomes, or the complete/nearly complete gene segment-2 sequences of several PBV strains from various host species, providing important insights into the genetic diversity and evolution of PBVs [1,2,10,13,14,15,16,17,18,19,20].

The small Indian mongoose (*Urva auropunctata*) is a predatory mammal that is native to the Middle East and southern Asia [21,22]. During the late 19th and early 20th centuries, these animals were introduced to the Caribbean islands to control rats and poisonous snakes [21]. Over time, the mongoose population increased to invasive levels on these islands, which is believed to have caused the extinction of several native wildlife species [21]. The small Indian mongoose poses a risk as a potential source of zoonotic pathogens to humans [21].

Although PBVs have been detected in a wide variety of host species including wildlife [5,6,10,13,15,20], there have been no reports on PBVs from the mongoose so far. In the present study, we report for the first time detection and molecular characterization of complete/nearly complete gene segment-2 (full length minus partial 3′- untranslated region (UTR)) of PBV strains from the small Indian mongoose (*Urva auropunctata*) on the Caribbean island of St. Kitts. In addition, we identified a novel PBV-like RdRp gene sequence that uses an alternative mitochondrial genetic code (that of mold or invertebrate) for translation.

## 2. Materials and Methods

### 2.1. Ethics Statement

This study was approved by the Institutional Animal Care and Use Committee (IACUC) of the Ross University School of Veterinary Medicine, St. Kitts and Nevis (Approved IACUC Protocol Number: 17.04.13, IACUC Protocol Title: Trapping and necropsy for mongoose microbial ecology study, Dated 13 April 2017).

### 2.2. Sampling

We received aliquots of non-diarrheic fecal samples from 82 small Indian mongooses that were trapped, euthanized, and necropsied for a microbial ecology study on the Caribbean island of St. Kitts in 2017. The samples were stored at −80 °C until further analyses.

### 2.3. Screening for PBVs

Extraction of viral RNA from fecal samples was performed using the QIAamp Viral RNA Mini Kit (Qiagen Sciences, MD, USA) following the manufacturer’s instructions. Samples were screened for PBVs in separate RT-PCR assays using either PBV GI, or PBV GII specific primers that target a short fragment (201 bp and 369 bp, respectively) of gene segment-2, as previously described [23].

### 2.4. Amplification of Complete/Nearly Complete Gene Segment-2 of Mongoose PBV GI Strains

A significant portion (~1200 bp, corresponding to nt 283-nt 1478 of gene segment-2 of prototype PBV GI strain PBV/Human/CHN/1-CHN-97/1997) of gene segment-2 of the mongoose PBV strains (strains PBV/Mongoose/KNA/M33/2017, PBV/Mongoose/KNA/M45/2017, PBV/Mongoose/KNA/ M46/2017, PBV/Mongoose/KNA/M58/2017, PBV/Mongoose/KNA/M67/2017 and PBV/Mongoose/ KNA/M72/2017) was amplified by two separate, overlapping RT-PCRs using published primers PBV 1.2FP and PicoB43, as well as primers PicoB25 and PBV 1.2RP (primer sequences are shown in supplementary material S1) [23,24]. The remaining 5′- portion of gene segment-2 of the 6 mongoose PBV strains, and the 3′- portion of gene segment-2 of a single mongoose PBV strain (strain M58) could be amplified using a modified non-specific primer-based amplification method with modifications as previously described (elaborated in supplementary material S2) [13,19]. A new reverse primer, designated as PBV-Con3 (5′-AAT GGT TTA CTG CAC CAT CTC-3′, nt 1665-nt 1644 of gene segment-2 of mongoose PBV strain M58), was designed from a short stretch of nt sequence that is conserved in the 3′-UTR of gene segment-2 of mongoose PBV strain M58 and PBV GI strains from other host species. Primer PBV-Con3 was used in combination with an internal forward primer (designed from the previously obtained ~1200 bp sequence of respective PBV strains) to amplify the remaining 3′- portion of gene segment-2 of the five other mongoose PBV strains.

### 2.5. Amplification of the Nearly Full-Length RdRp Genes of a Novel PBV-Like Strain and a PBV GI Strain from the Same Fecal Sample

Although the PBV GI screening primers (PicoB25 and PicoB43), and the primer combination PicoB25 and PBV 1.2RP amplified a PBV GI strain (strain PBV/Mongoose/KNA/M17B/2017), primers PBV 1.2FP and PicoB43 unexpectedly amplified a novel PBV-like strain (designated as strain PBV/Mongoose/KNA/M17A/2017) in the same fecal sample, as revealed by sequencing of the PCR amplicons. Internal primers designed from the already obtained M17A and M17B sequences were used in combination with the modified non-specific primer-based amplification method and conventional RT-PCRs to obtain the nearly full-length gene segment-2/RdRp gene sequence for respective strains. All the steps (RNA extraction to sequencing) were repeated thrice to validate the mixed infection in sample M17.

### 2.6. Nucleotide Sequencing

The PCR products were purified using the Wizard^®^ SV Gel and PCR Clean-Up kit (Promega, WI, USA) according to manufacturers’ instructions. Nucleotide sequences were determined using the ABI Prism Big Dye Terminator Cycle Sequencing Ready Reaction Kit (Applied Biosystems, CA, USA) on an ABI 3730XL Genetic Analyzer (Applied Biosystems, CA, USA.).

### 2.7. Sequence Analysis

Open reading frames (ORFs) were determined using the ORF finder (https://www.ncbi.nlm.nih.gov/orffinder/). Homology search for related cognate nt and deduced aa sequences were performed using the standard BLASTn program and BLASTp program, respectively (Basic Local Alignment Search Tool, www.ncbi.nlm.nih.gov/blast). Multiple alignments of nt and deduced aa sequences were performed using the CLUSTALW program (version ddbj, http://clustalw.ddbj.nig.ac.jp/) with default parameters. The phylogenetic tree was created by the Maximum Likelihood (ML) method using the MEGA6 software, and statistically supported by 500 bootstrap replicates [25]. Phylogenetic distances were measured using the LG + G model of substitution, as described previously [20]. To rule out biases in clustering patterns, phylogenetic analysis was repeated using other mathematical models, such as the Poisson model and JTT model.

### 2.8. GenBank Accession Numbers

The GenBank accession numbers for the complete/nearly complete nt sequences of gene segment-2/RdRp gene of the mongoose PBV and PBV-like strains are MN563295-MN563302.

## 3. Results and Discussion

**Figure 1:**
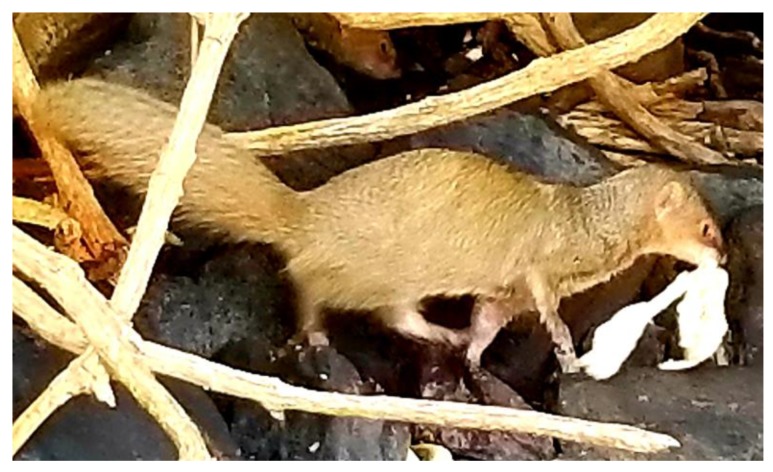
The small Indian mongoose (*Urva auropunctata*) on the Caribbean island of St. Kitts.

The federation of St. Kitts and Nevis is a twin island country in the Caribbean region. Although there are no official estimates of the small Indian mongoose population on St. Kitts and Nevis, it is believed to be over 45,000 [26] (Figure 1). In St. Kitts and Nevis, the free-roaming mongoose thrives in wild, rural, and urban habitats [26].

### 3.1. Detection of PBVs in Mongooses

**Figure 2:**
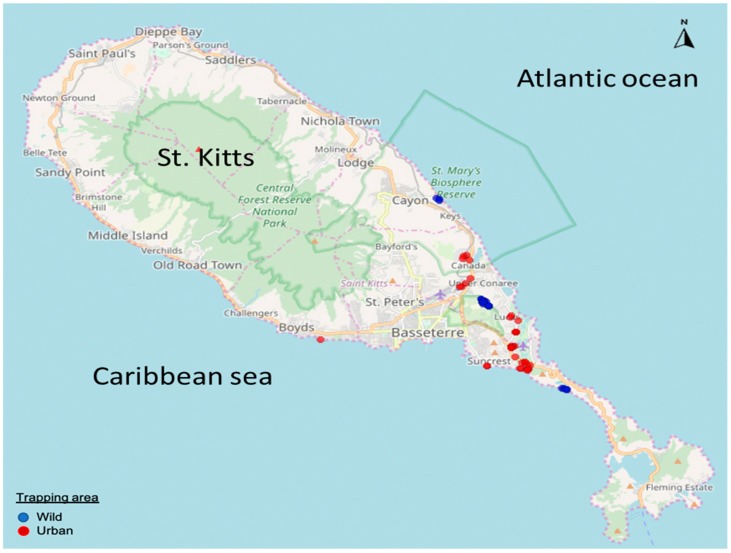
Map of island of St. Kitts showing the mongoose trapping sites. The trapping sites in wild and urban habitats are highlighted with blue and red, respectively.

In the present study, we reported high rates of detection (35.36%, 29/82) of GI PBVs in fecal samples collected from the small Indian mongoose on St. Kitts. None of the samples tested positive for GII PBVs. The screening results were confirmed by sequencing the ~201 bp PCR amplicons of gene segment-2 of the mongoose PBV GI strains. None of the PBV positive mongooses exhibited clinical signs of diarrhea, corroborating previous reports on asymptomatic PBV infection in wildlife [5,6,13,15]. The mongooses were trapped in both urban and wild habitats, and most likely came into contact with humans and various animal species (cats, dogs, livestock, rodents, and vervet monkeys), which may have increased their chances of exposure and PBV infection (Figure 2). The rate of detection of PBVs in mongooses from the urban and wild habitats was 33.33% (19/57) and 40% (10/25), respectively.

### 3.2. Analysis of Partial Gene Segment-2 of Mongoose PBV GI Strains

In the present study, the partial gene segment-2 sequences (<201 nt) of 16 PBV GI strains were of sufficient quality to obtain preliminary insights into the genetic diversity of mongoose PBVs on St. Kitts. The partial putative RdRp sequences of the mongoose PBV GI strains shared deduced aa identities of 49%–100% between themselves, and exhibited maximum identities of 65%–100% with cognate PBV sequences from various other host species/environmental samples, revealing high genetic diversity of the mongoose PBVs within and between host species (Supplementary material S3). Although BLASTn analysis of the remaining partial gene segment-2 sequences revealed maximum homology with PBV GI strains, the sequence data lacked quality, which may indicate amplification of multiple different PBV strains in the same sample. Since the complete gene segment-2 of PBV is ~1700 bp, analysis of the partial mongoose PBV sequences was considered inconclusive.

### 3.3. Molecular Characterization of Complete/Nearly Complete Gene Segment-2 of Mongoose PBV GI Strains

Based on available volumes of fecal samples and the quality of partial nt sequences, only seven mongoose PBV GI strains qualified for molecular characterization of the complete/nearly complete (full-length minus partial 3′-UTR) gene segment-2. The complete/nearly complete gene segment-2 of the mongoose PBV GI strains was amplified by combining a modified non-specific primer-based amplification method with conventional RT-PCRs. Since the non-specific primer method is difficult, time consuming and requires large volumes of viral RNA as starting material [13,14,15,16,19], the inclusion of a newly designed reverse primer, PBV-Con3, greatly facilitated the amplification of the almost complete 3′- end region of the PBV strains by conventional RT-PCRs.

**Figure 3:**
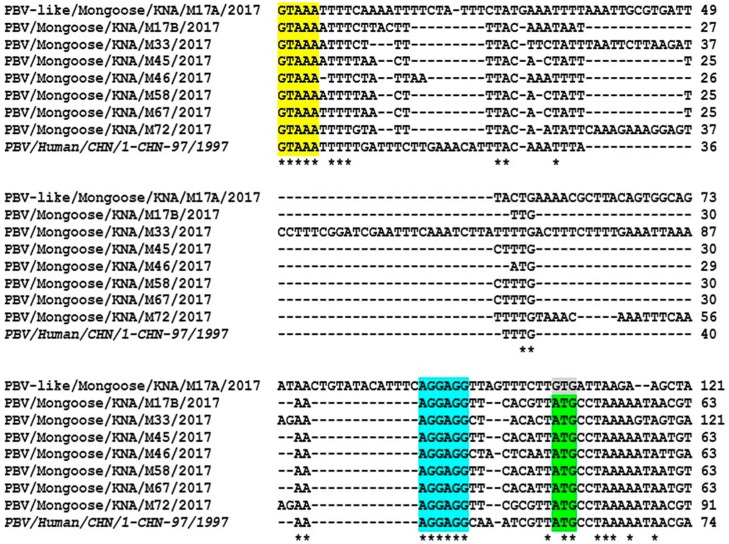
Alignment of the 5′- region of the mongoose PBV and PBV-like RNA-dependent RNA polymerase (RdRp) gene sequences with that of prototype PBV GI strain PBV/Human/CHN/1-CHN-97/1997 (indicated with *italic type*). The 5′- terminal sequence (highlighted with yellow) and the bacterial ribosomal binding site (RBS) sequence (highlighted with blue) that are conserved in gene segment-2 of PBVs were retained in the mongoose PBV and PBV-like RdRp sequences. The putative start codon (ATG) for RdRp in gene segment-2 of mongoose PBV GI strains has been highlighted with green, whilst the putative alternative initiation codon (GTG) for RdRp in the novel PBV-like sequence (M17A) is shown with gray. A ‘*’denotes an identical nucleotide (nt) residue, whilst ‘-’ indicates absence of nt residue. Numbers to the right indicate the positions of the nt for respective PBV strains. Alignment of the complete/nearly complete mongoose PBV and PBV-like RdRp gene sequences with that of the prototype PBV GI strain is shown in supplementary material S4.

The features of the complete/nearly complete gene segment-2 of the 7 mongoose PBV GI strains are shown in Figure 3, Table 1, and supplementary material S4. The 5′- terminal nt sequence (GUAAA) of PBV gene segment-2 has been proposed to be crucial for initiation of virus replication, and is conserved in PBVs from various host species [1,27]. The 5′- end of gene segment-2 of the mongoose PBV GI strains retained the ‘GUAAA’ sequence (Figure 3). Based on the identification of a classically defined prokaryotic motif (the ribosomal binding site (RBS) sequence, AGGAGG) in the 5′-UTR of gene segments -1 and -2 of all published PBV strains, a recent study has proposed that PBVs are prokaryotic viruses [7]. Interestingly, the 5′-UTR of gene segment-2 of all the mongoose PBV GI strains retained the RBS sequence upstream of the putative start codon for the RdRp gene, corroborating the hypothesis (Figure 3). The 3′- end sequence (ACUGC) of gene segment-2 that is conserved in PBVs was present in mongoose PBV strain M58 [1], whilst the 3′- terminal sequences for gene segment-2 of the remaining mongoose PBV strains were not determined in this study (Supplementary material S4).

**Figure 4:**
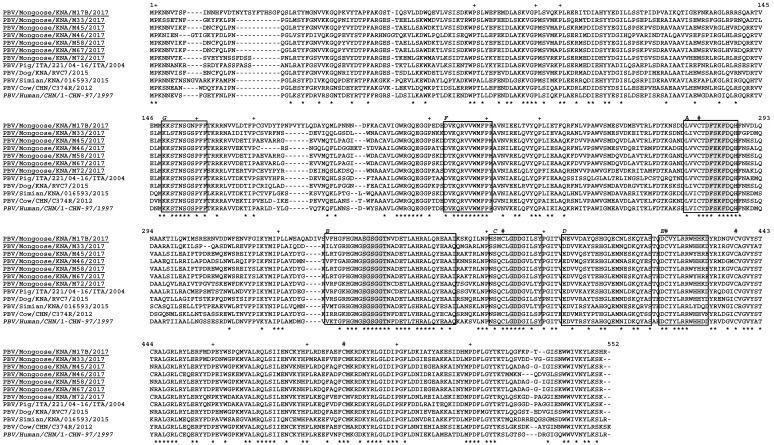
Alignment of the complete deduced amino acid (aa) sequences of putative RNA-dependent RNA polymerases (RdRps) of picobirnavirus (PBV) genogroup-I (GI) strains detected in the small Indian mongoose on the Caribbean island of St. Kitts with those of PBV strains from other host species using the ClustalW program (http://clustalw.ddbj.nig.ac.jp/, accessed 15 October 2019). The PBV strains from mongoose are highlighted with underlines, whilst the prototype GI strain PBV/Human/CHN/1-CHN-97/1997 is indicated by *italic type*. The three conserved domains in the putative RdRps are highlighted with gray, whilst the seven polymerase motifs (G, F, A, B, C, D, and E) are shown with boxes [27]. Conserved cysteine and proline residues are shown by ‘#’ and ‘+’, respectively. A ‘*’ denotes an identical aa residue, whilst ‘-’ indicates absence of an aa residue. Positions of aa residues correspond to those of PBV strain PBV/Mongoose/KNA/M17B/2017.

A single putative ORF encoding the viral RdRp was present in gene segment-2 of the mongoose PBV GI strains (Table 1, supplementary material S4). The lengths of the putative RdRps of the mongoose PBV strains ranged from 528 aa to 552 aa, which was 2 aa less and 22 aa longer than that of prototype PBV GI strain 1-CHN-97, respectively (Table 1). The multiple alignment of the putative RdRps of the mongoose PBV GI strains with those of PBV strains from other host species including the prototype GI strain is shown in Figure 4. The three motifs (DFXKFD, SGSGGT, and GDD) that are conserved in the putative RdRps of PBVs from various host species were retained in the deduced aa sequences of gene segment-2 of the mongoose PBV GI strains (Figure 4) [1,13,14,15,16,19,23].

**Figure 5:**
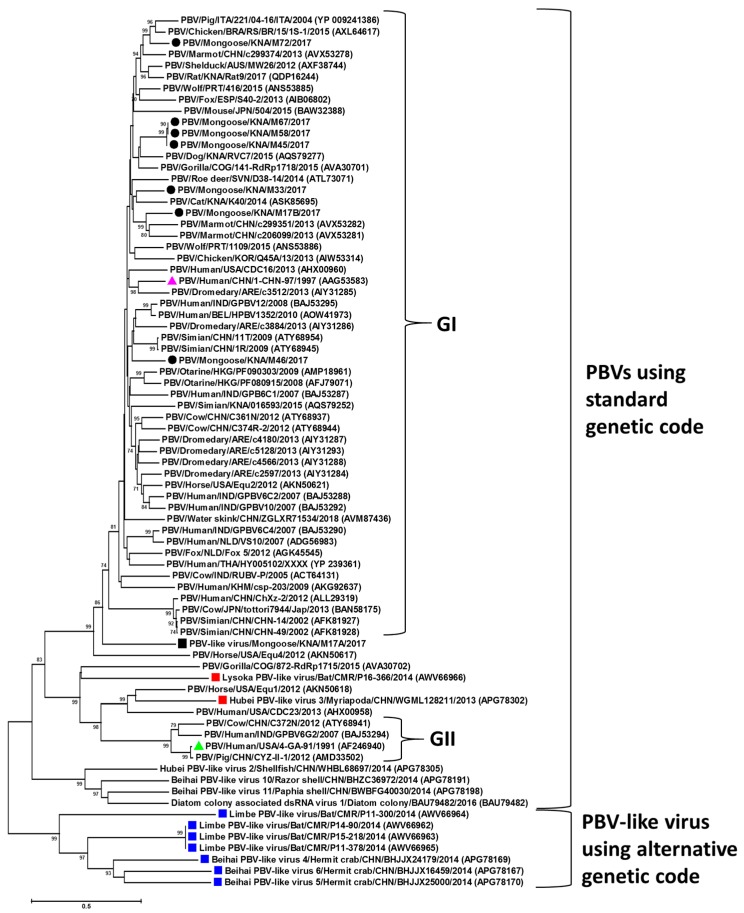
Phylogenetic analysis of the putative RNA-dependent RNA polymerases of the mongoose picobirnavirus (PBV) genogroup-I (GI) strains and the novel mongoose PBV-like virus with those of PBVs that use the standard genetic code and PBV-like viruses that use an alternative mitochondrial genetic code. GenBank accession numbers are shown in parentheses. Bootstrap values < 70% are not shown. Scale bar, 0.5 substitutions per amino acid. Black circles: mongoose PBV GI strains; Black square: the novel mongoose PBV-like virus that uses an alternative mold mitochondrial genetic code for translation; pink triangle: prototype PBV GI strain; green triangle: prototype PBV GII strain; blue squares: PBV-like viruses that use alternative genetic code and cluster separately from PBVs using the standard genetic code; red squares: PBV-like strains that use alternative genetic code, yet cluster within PBVs using standard genetic code.

The maximum deduced aa identities of the putative RdRps of the mongoose PBV GI strains between themselves and with those of PBVs from other host species are shown in Table 1. The deduced aa identities between the putative RdRps of the mongoose PBV strains were in the range of 59%–99%, with strains M45, M58, and M67 sharing 99% sequence identities with each other (Table 1). The gene segment-2 of the mongoose PBV strains shared low deduced aa identities with those of PBV strains from other host species (Table 1). These observations were corroborated by phylogenetic analysis of the putative RdRps, where the mongoose PBVs were distributed within the GI cluster (Figure 5). Mongoose PBV strains M45, M58, and M67 formed a single cluster, distinct from other PBV strains (Figure 5). On the other hand, mongoose PBV strains M17B, M33, M46, and M72 formed separate branches within the PBV GI cluster (Figure 5).

### 3.4. Identification of a Novel PBV-Like RdRp Gene Sequence That Uses an Alternative Genetic Code for Translation

In the present study, a novel PBV-like RdRp gene sequence (M17A) that uses an alternative mitochondrial genetic code for translation was detected by accident during amplification of the complete gene segment-2 of mongoose PBV GI strain M17B. Although the nt sequence of PBV-like strain M17A retained the conserved features seen in 5′-UTR of gene segment-2 of other PBVs, it lacked the putative ORF for RdRp using the standard genetic code (Figure 3, supplementary material S5). Surprisingly, a single large ORF (1533 nt) encoding the putative RdRp (510 aa) was identified using both the mold mitochondrial genetic code and the invertebrate mitochondrial genetic code (Figure 3, Table 1, supplementary material S6 and S7).

The putative RdRp of PBV-like strain M17A was 20 aa shorter than that of the prototype PBV GI strain (supplementary material S8). The M17A RdRp exhibited a maximum deduced aa identity of 51% with a PBV GI strain from marmot (GenBank accession number AVX53249) (Table 1). Phylogenetically, the putative RdRp of M17A clustered near PBV GI strains (Figure 5). The three motifs (DFXKFD, SGSGGT, and GDD) that are conserved in PBVs were present in the putative RdRp of M17A (supplementary material S8).

Recently, novel PBV-like RdRp gene sequences that use an alternative mitochondrial genetic code have been detected in bats, crustaceans and myriapods [10,11]. Phylogenetically, PBV-like RdRp sequences using the alternative genetic code group into a single clade that is distinct from the PBV RdRp clade using the standard genetic code (Figure 5) [10]. Based on analysis of RdRp sequences that belong to the clade of PBV-like viruses using the alternative genetic code, and the lack of identifiable PBV-like capsid sequences in the respective metagenomics data, Yinda et al. (2018) proposed that PBV-like strains using the invertebrate mitochondrial genetic code might behave like mitoviruses [10]. Mitoviruses are virus-like elements that are omnipresent in the mitochondria of fungi, possess a plus-strand RNA genome that generally employ the mold mitochondrial genetic code to translate RdRp, and lack a capsid [9,28,29].

On the other hand, at least two PBV-like strains (strain Lysoka_picobirna-like_virus_P16-366 from a bat and strain WGML128211_ Hubei_picobirna-like_virus_3 from a myriapod) that use the alternative mitochondrial genetic code were found to cluster within PBVs using the standard genetic code, and a capsid sequence has been identified for PBV-like strain P16-366 (Figure 5) [10]. Recently, Wolf et al. (2018) proposed that dsRNA viruses of the partitivirus-picobirnavirus group may have evolved through reassortment of genomic segments encoding, respectively, a plus-strand RNA virus RdRp (possibly derived from a naked RNA replicon) and a dsRNA virus capsid protein related to those of the major cluster of dsRNA viruses that include cystoviruses, reoviruses and totiviruses [9]. This hypothesis might offer a plausible explanation for the possible origin of PBV-like strain P16-366.

Considering the above, the mongoose PBV-like RdRp sequence (M17A) appeared to be intriguing, as M17A retained the conserved features of PBV gene segment-2/RdRps, and clustered near PBV GI strains within the clade of PBVs that use the standard genetic code, whilst using an alternative mitochondrial genetic code (especially that of mold) for translation (Figure 5). Although we could not determine the presence or absence of a capsid sequence for M17A, our observations together with the: (1) hypotheses of Wolf et al. (2018) and Yinda et al. (2018), (2) the inclusion in the partitivirus-picobirnavirus clade of some naked RNA replicons that reproduce in algal mitochondria or chloroplasts, use a mitochondrial genetic code, and behave like mitoviruses, and (3) identification of the conserved prokaryotic RBS sequence (Shine-Dalgarno sequence) in PBV genomes including the mongoose PBV and PBV-like strains provided evidence that PBVs may infect prokaryotic or fungal host cells [7,8,9,10].

### 3.5. Conclusions

To our knowledge, this is the first report on the detection of PBVs in mongooses. Although we observed high rates of detection of PBVs in mongooses with the widely used PBV GI screening primers, it should be noted that the GI and GII primer sequences used in this study were published in 2000 [23]. Considering the current genetic diversity of PBVs, these screening primers may not be representative of the family *Picobirnaviridae*.

Molecular characterization of the complete/nearly complete gene segment-2 of the mongoose PBVs provided first time insights into the genetic make-up and various features of gene segment-2/putative RdRps of PBVs from a new animal species. The mongoose PBVs exhibited high genetic diversity among themselves and with PBVs from various other host species, which was similar to those observed in previous studies [1,5,6,13,14,15,16,30]. Considering the possibility of interspecies transmission and reassortment events involving PBVs [5,6,20,30], and high rates of detection of PBVs in the small Indian mongoose, it would be interesting to conduct further studies on PBVs in different species of the mongoose.

The presence of the prokaryotic RBS sequence in the mongoose PBV genomes, and analysis of a novel mongoose PBV-like RdRp gene sequence that uses an alternative genetic code (especially that of mold mitochondria) for translation corroborated recent speculations that PBVs may actually infect prokaryotic or fungal host cells, warranting further studies in identifying the true host/s of PBVs [7,8,9,10].

## Figures and Tables

**Table 1:** Features of gene segment-2, and deduced amino acid (aa) identities of the putative RNA-dependent RNA polymerase (RdRp) of picobirnavirus (PBV) and PBV-like strains detected in the small Indian mongoose on the Caribbean island of St. Kitts.

Strain	Length of Nucleotide (nt) Sequence Obtained	Putative Open Reading Frame Encoding RdRp	Length of Putative RdRp	Maximum Deduced aa Identity (%) of Putative RdRp	
				With Other Mongoose PBV Strains ^1^	With PBV Strains from Other Host Species ^2^, (*GenBank Accession Number*)
PBV-like/Mongoose/KNA/M17A/2017	1662 nt ^3^	nt 107-nt 1639 ^4^	510 aa ^4^	**48%** with PBV/Mongoose/KNA/M33/2017 and PBV/Mongoose/KNA/M45/2017	**51%** with PBV/Marmot/CHN/c159756/2013, (*AVX53249*)
PBV/Mongoose/KNA/M17B/2017	1715 nt ^3^	nt 47-nt 1705	552 aa	**64%** with PBV/Mongoose/KNA/M33/2017	**69%** with PBV/Marmot/CHN/c206099/2013, (*AVX53281*)
PBV/Mongoose/KNA/M33/2017	1701 nt ^3^	nt 105-nt 1691	528 aa	**68%** with PBV/Mongoose/KNA/M45/2017, PBV/Mongoose/KNA/M58/2017 and PBV/Mongoose/KNA/M67/2017	**71%** with PBV/Cat/KNA/K40/2014, (*ASK85695*)
PBV/Mongoose/KNA/M45/2017	1651 nt ^3^	nt 47-nt 1639	530 aa	**99%** with PBV/Mongoose/KNA/M58/2017 and PBV/Mongoose/KNA/M67/2017	**78%** with PBV/Gorilla/COG/141-RdRp1718/2015, (*AVA30701*)
PBV/Mongoose/KNA/M46/2017	1650 nt ^3^	nt 47-nt 1636	529 aa	**64%** with PBV/Mongoose/KNA/M33/2017	**72%** with PBV/Simian/CHN/1R/2009, (*ATY68945*)
PBV/Mongoose/KNA/M58/2017	1689 nt ^5^	nt 47-nt 1639	530 aa	**99%** with PBV/Mongoose/KNA/M45/2017 and PBV/Mongoose/KNA/M67/2017	**77%** with PBV/Gorilla/COG/141-RdRp1718/2015, (*AVA30701*)
PBV/Mongoose/KNA/M67/2017	1654 nt ^3^	nt 47-nt 1639	530 aa	**99%** with PBV/Mongoose/KNA/M45/2017 and PBV/Mongoose/KNA/M58/2017	**77%** with PBV/Gorilla/COG/141-RdRp1718/2015, (*AVA30701*)
PBV/Mongoose/KNA/M72/2017	1691 nt ^3^	nt 75-nt 1679	534 aa	**65%** with PBV/Mongoose/KNA/M33/2017	**81%** with PBV/Chicken/BRA/RS/BR/15/1S-1/2015, (*AXL64617*)

^1, 2^ Deduced aa identities were determined using the ClustalW ^1^ program (http://clustalw.ddbj.nig.ac.jp/, accessed 22 November 2019), and the BLASTp. ^2^ program (https://blast.ncbi.nlm.nih.gov/Blast.cgi?PAGE=Proteins, accessed 22 November 2019). ^3^ Nearly complete nt sequence of gene segment-2 (full-length sequence minus partial 3’-untranslated region). ^4^ The nt sequence of PBV-like/Mongoose/KNA/M17A/2017 lacked a putative RdRp open reading frame using the standard genetic code, and used an alternative mitochondrial genetic code (that of mold or invertebrate) for translation (Shown in supplementary materials S5, S6 and S7). The putative RdRp obtained using the mold mitochondrial code was used for determining aa identities. ^5^ Complete nt sequence of gene segment-2.

## Supplementary Materials

The following are available online at https://www.mdpi.com/1999-4915/12/1/99/s1, Supplementary material S1: Primers used for amplification of a significant portion (~1200 bp) of gene segment-2 of the mongoose picobirnavirus (PBV) strains, Supplementary material S2: Amplification of the 5′- and 3′- regions of gene segment-2/RdRp genes of the mongoose picobirnavirus (PBV) and PBV-like strains using a modified non-specific primer-based amplification method [13,19], Supplementary material S3: GenBank accession numbers of partial gene segment-2 sequences (<201 bp) and deduced amino acid (aa) identities of the partial putative RNA-dependent RNA polymerases (RdRp) of picobirnavirus (PBV) strains detected in the small Indian mongoose on the Caribbean island of St. Kitts, Supplementary material S4: Alignment of the complete/nearly complete nucleotide (nt) sequences (full-length minus partial 3′-UTR) of gene segment-2/RNA-dependent RNA polymerase (RdRp) genes of picobirnavirus (PBV) and PBV-like strains detected in the small Indian mongoose on the Caribbean island of St. Kitts with that of prototype PBV genogroup-I strain PBV/Human/CHN/1-CHN-97/1997, Supplementary material S5: The three translation frames (5′-3′) of the novel mongoose PBV-like sequence (designated as M17A) using the standard genetic code, Supplementary material S6: Translation (5′-3′, frame 2) of the novel mongoose PBV-like sequence M17A using an alternative mold mitochondrial genetic code, Supplementary material S7: Translation (5′-3′, frame 2) of the novel mongoose PBV-like sequence M17A using an alternative invertebrate mitochondrial genetic code, Supplementary material S8: Alignment of the putative RNA-dependent RNA polymerase (RdRp) of the novel mongoose PBV-like strain M17A with those of the prototype human genogroup-I PBV strain (PBV/Human/ CHN/1-CHN-97/1997) and mongoose PBV GI strain PBV/Mongoose/KNA/M17B/2017.
