# Supplementary material for: Detection and Molecular Characterization of Picobirnaviruses (PBVs) in the Mongoose: Identification of a Novel PBV Using an Alternative Genetic Code
Source: Viruses. 2020 Jan 15;12(1):99. doi: 10.3390/v12010099 (PMC7019992; doi:10.3390/v12010099)
Supplement: Supplementary file 1 [file viruses-12-00099-s001.zip › Supplementary material S1.pdf]

**Supplementary material S1.** Primers used for amplification of a significant portion (~1200 bp) of gene segment-2 of the mongoose picobirnavirus (PBV) strains.

| Primer name | Sequence (5'- 3') | Nucleotide (nt) position corresponding to gene segment-2 of prototype PBV genogroup-I strain 1-CHN-97 | Reference |
|---|---|---|---|
| PBV 1.2FP | AAG GTC GGK CCR ATG T | nt 283- nt 298 | 24 |
| PBV 1.2RP | TTA TCC CYT TTC ATG CA | nt 1478-nt 1462 | 24 |
| PicoB25 | TGG TGT GGA TGT TTC | nt 665-nt 679 | 23 |
| PicoB43 | ART GYT GGT CGA ACT T | nt 865-nt 850 | 23 |
