# Supplementary material for: Detection and Molecular Characterization of Picobirnaviruses (PBVs) in the Mongoose: Identification of a Novel PBV Using an Alternative Genetic Code
Source: Viruses. 2020 Jan 15;12(1):99. doi: 10.3390/v12010099 (PMC7019992; doi:10.3390/v12010099)
Supplement: Supplementary file 1 [file viruses-12-00099-s001.zip › Supplementary material S2.pdf]

Supplementary material S2. *Amplification of the 5′- and 3′- regions of gene segment-2/RdRp genes of the mongoose picobirnavirus (PBV) and PBV-like strains using a modified non-specific primer-based amplification method [13, 19].*

A single amino group-linked oligonucleotide primer (primer A, 5′- CCC TCG AGT ACT AAC TAG TTA ACT GAT CAC CTC TAG ACC TTT -3′, 5′- end phosphorylated and 3′- end incorporated an NH2 blocking group) was ligated to the 3'- ends of both strands of double-strand RNA (dsRNA) of the mongoose PBV and PBV-like strains using T4 RNA ligase (Takara Bio Inc., Japan) following the instructions of the manufacturer. After 17 hours of incubation at 8oC, the MinElute Gel Extraction Kit (Qiagen Sciences, USA) was used to purify and concentrate the ligated dsRNA. Thereafter, the 5'- region of gene segment-2 of the mongoose PBV and PBV-like strains and the 3'- portion of gene segment-2 of mongoose PBV strain M58 was amplified using an internal reverse primer and an internal forward primer (designed from the already obtained ~1200 bp gene segment-2 sequence of respective PBV/PBV-like strains), respectively, and a common end primer C (5′- GGT CTA GAG GTG ATC AGT TAA CTA GTT AGT ACT -3′, complementary to primer-A) by the QIAGEN OneStep RT-PCR Kit (Qiagen Biosciences, USA) following manufacturer's instructions.
