# Supplementary material for: Detection and Molecular Characterization of Picobirnaviruses (PBVs) in the Mongoose: Identification of a Novel PBV Using an Alternative Genetic Code
Source: Viruses. 2020 Jan 15;12(1):99. doi: 10.3390/v12010099 (PMC7019992; doi:10.3390/v12010099)
Supplement: Supplementary file 1 [file viruses-12-00099-s001.zip › Supplementary material S3.pdf]

Supplementary material S3. GenBank accession numbers of partial gene segment-2 sequences (<201 bp) and deduced amino acid (aa) identities of the partial putative RNA-dependent RNA polymerase (RdRp) sequences of picobirnavirus (PBV) strains detected in the small Indian mongoose on the Caribbean island of St. Kitts.

Mongoose PBV strain [1]	GenBank accession number	Maximum/significant deduced aa identity [2] with PBVs from other host species/sources (*GenBank accession number*)
PBV/Mongoose/KNA/M10/2017	MN729472	90% with human strain PBV/Human/CMRHP25A/CMR/2014 (*QAA77624*)
PBV/Mongoose/KNA/M15/2017	MN729473	84% with guinea pig PBV strain GP6/India/2017 (*AUB54549*)
PBV/Mongoose/KNA/M21/2017	MN729474	100% with PBV strain from raw sewage sample (*ACL13389*)
PBV/Mongoose/KNA/M26/2017	MN729475	89% with PBV strain from raw sewage sample (*ACL13497*)<br>87% with porcine PBV strain CHHN-B7 (*AHM01745*)
PBV/Mongoose/KNA/M31/2017	MN729476	65% with porcine PBV strain PBV_ segment_ S_ VIRES_ YN01_ C2 (*QBJ01804*)
PBV/Mongoose/KNA/M38/2017	MN729477	90% with canine PBV strain dog/BR-02/BRA/2004 (*ACN25100*)
PBV/Mongoose/KNA/M49/2017	MN729478	87% with murine PBV strain MPV3/NYC/Manhattan/poolP30 (*AWB14662*)
PBV/Mongoose/KNA/M74/2017	MN729479	91% with porcine PBV strain PoPBV_ segment_ S_ VIRES_ NM02_C2 (*QBJ01787*)
PBV/Mongoose/KNA/M83/2017	MN729480	83% with canine PBV strain dog/BR-02/BRA/2004 (*ACN25100*)

[1] Mongoose PBV strains that were molecularly characterized for the complete/nearly complete gene segment-2 are not shown here.

[2] Deduced aa identities were determined using the BLASTp software (https://blast.ncbi.nlm.nih.gov/Blast.cgi, accessed 25th November 2019)
