# Supplementary material for: Detection and Molecular Characterization of Picobirnaviruses (PBVs) in the Mongoose: Identification of a Novel PBV Using an Alternative Genetic Code
Source: Viruses. 2020 Jan 15;12(1):99. doi: 10.3390/v12010099 (PMC7019992; doi:10.3390/v12010099)
Supplement: Supplementary file 1 [file viruses-12-00099-s001.zip › Supplementary material S4.pdf]

Supplementary material S4. Alignment of the complete/nearly complete nucleotide (nt) sequences (full-length minus partial 3'-UTR) of gene segment-2/RNA-dependent RNA polymerase (RdRp) genes of picobirnavirus (PBV) and PBV-like strains detected in the small Indian mongoose on the Caribbean island of St. Kitts with that of prototype PBV genogroup-I strain PBV/Human/CHN/1-CHN-97/1997. A '*' denotes an identical nt residue, whilst '-' indicates absence of a nt residue. The 5'- terminal sequence, the bacterial ribosomal binding site (RBS) sequence and the 3'- terminal sequence that are conserved in PBVs is shown with yellow, blue and pink, respectively. The putative start codon (ATG) and stop codon (TAA) for the putative RdRp gene of mongoose PBV GI strains is highlighted with green and red, respectively, whilst those of PBV-like strain PBV-like/Mongoose/KNA/M17A/2017 is shown with grey and teal, respectively. Numbers to the right indicate the positions of the nt for respective PBV strains.

```
PBV-like/Mongoose/KNA/M17A/2017    GTAAATTTTCAAAATTTTCTA-TTTCTATGAAATTTTAAATTGCGTGATT 49
PBV/Mongoose/KNA/M17B/2017         GTAAAATTTCTTACTT---------TTAC-AAATAAT------------- 27
PBV/Mongoose/KNA/M33/2017          GTAAAATTTCT---TT---------TTAC-TTCTATTTAATTCTTAAGAT 37
PBV/Mongoose/KNA/M45/2017          GTAAAATTTTAA--CT---------TTAC-A-CTATT------------T 25
PBV/Mongoose/KNA/M46/2017          GTAAA-TTTCTA--TTAA-------TTAC-AAATTTT------------- 26
PBV/Mongoose/KNA/M58/2017          GTAAAATTTTAA--CT---------TTAC-A-CTATT------------T 25
PBV/Mongoose/KNA/M67/2017          GTAAATTTTTAA--CT---------TTAC-A-CTATT------------T 25
PBV/Mongoose/KNA/M72/2017          GTAAATTTTGTA--TT---------TTAC-A-ATATTCAAAGAAAGGAGT 37
PBV/Human/CHN/1-CHN-97/1997        GTAAATTTTTGATTTCTTGAAACATTTAC-AAATTTA------------- 36
                                   ***** ***                 **     *

PBV-like/Mongoose/KNA/M17A/2017    --------------------------TACTGAAAACGCTTACAGTGGCAG 73
PBV/Mongoose/KNA/M17B/2017         ----------------------------TTG------------------- 30
PBV/Mongoose/KNA/M33/2017          CCTTTCGGATCGAATTTCAAATCTTATTTTGACTTTCTTTTGAAATTAAA 87
PBV/Mongoose/KNA/M45/2017          --------------------------CTTTG------------------- 30
PBV/Mongoose/KNA/M46/2017          ----------------------------ATG------------------- 29
PBV/Mongoose/KNA/M58/2017          --------------------------CTTTG------------------- 30
PBV/Mongoose/KNA/M67/2017          --------------------------CTTTG------------------- 30
PBV/Mongoose/KNA/M72/2017          --------------------------TTTTGTAAAC-----AAATTTCAA 56
PBV/Human/CHN/1-CHN-97/1997        ---------------------------TTTG------------------- 40
                                                                **

PBV-like/Mongoose/KNA/M17A/2017    ATAACTGTATACATTTCAGGAGGTTAGTTTCTTGTGATTAAGA--AGCTA 121
PBV/Mongoose/KNA/M17B/2017         --AA-------------AGGAGGTT--CACGTTATGCCTAAAAATAACGT 63
PBV/Mongoose/KNA/M33/2017          AGAA-------------AGGAGGCT---ACACTATGCCTAAAAGTAGTGA 121
PBV/Mongoose/KNA/M45/2017          --AA-------------AGGAGGTT--CACATTATGCCTAAAAATAATGT 63
PBV/Mongoose/KNA/M46/2017          --AA-------------AGGAGGCTA-CTCAATATGCCTAAAAATATTGA 63
PBV/Mongoose/KNA/M58/2017          --AA-------------AGGAGGTT--CACATTATGCCTAAAAATAATGT 63
PBV/Mongoose/KNA/M67/2017          --AA-------------AGGAGGTT--CACATTATGCCTAAAAATAATGT 63
PBV/Mongoose/KNA/M72/2017          AGAA-------------AGGAGGTT--CGCGTTATGCCTAAAAATAACGT 91
PBV/Human/CHN/1-CHN-97/1997        --AA-------------AGGAGGCAA-ATCGTTATGCCTAAAAATAACGA 74
                                     **             ******         * **  *** *  *

PBV-like/Mongoose/KNA/M17A/2017    AACT-------TGAAGGACTAT------------TTCATCC----TTC-C 147
PBV/Mongoose/KNA/M17B/2017         AACA--AGTT-TTATTAATAACCACTTCGTGGATACTAAC-----TACAC 105
PBV/Mongoose/KNA/M33/2017          AACT--AATT-TTGGTAAGTAT-----------TTTAAAC-----TAC-C 151
PBV/Mongoose/KNA/M45/2017          AATT--AAAT-TCGATAATTGC-----------TTTCAAT-----TAC-C 93
PBV/Mongoose/KNA/M46/2017          AAACGGAACTATTGGTAAATAT------------TTCGATT----TAC-C 96
PBV/Mongoose/KNA/M58/2017          AATT--AAAT-TCGATAATTGC-----------TTTCAAT-----TAC-C 93
PBV/Mongoose/KNA/M67/2017          AATT--AAAT-TCGATAATTGC-----------TTTCAAT-----TAC-C 93
PBV/Mongoose/KNA/M72/2017          AACT--AAGT-CAGTCGAGTAC-----------TA-CAATAGTAGTTT-C 125
PBV/Human/CHN/1-CHN-97/1997        AGTT--AGTT-TTGGTGAGTAC------------TTTAACT----TAC-C 104
                                   *                *                           *   *
```

Supplementary material S4 (contd.)

```
PBV-like/Mongoose/KNA/M17A/2017  CAATCAG------------GGTTT---AC------AGTCTTATTTACACA 176
PBV/Mongoose/KNA/M17B/2017       GAGTTACTTCACACATAGTGGTTTCCAATCACTTCGGACTTATATGGGCA 155
PBV/Mongoose/KNA/M33/2017        AAATCCG------------GGATT--AAT-------GTCCTATTTTGGCA 180
PBV/Mongoose/KNA/M45/2017        GAATCAA------------GGTTT--AA-------GGACGTATTTTGGCA 122
PBV/Mongoose/KNA/M46/2017        AAACCCT------------GGACT--GGT-------GTCATATTTTGACA 125
PBV/Mongoose/KNA/M58/2017        GAATCAA------------GGTTT--AA-------GGACGTATTTTGGCA 122
PBV/Mongoose/KNA/M67/2017        GAATCAA------------GGTTT--AA-------GGACGTATTTTGGCA 122
PBV/Mongoose/KNA/M72/2017        GACTCAA------------GTCCT--AACT--TACGGACGTATTTTGGCA 159
PBV/Human/CHN/1-CHN-97/1997      TAATCCG------------GGACT--AA-------AGTCCTATTTTGACA 133
                                  *                 *   *            * * *** *   **

PBV-like/Mongoose/KNA/M17A/2017  AGACGGAGGTAGGTAGCGACCTTGACATTCGT---ACTCCGTTTT---GG 220
PBV/Mongoose/KNA/M17B/2017       ATGTCGTTAAAGG--ACAGC-CTAAAGTCTACACAGCTCCATTCGCCAAG 202
PBV/Mongoose/KNA/M33/2017        ATGTCAAGAAAGG--ACAAC-CCAACATTTATGACACACCTTTCGCCAGA 227
PBV/Mongoose/KNA/M45/2017        ACGTCGTTAAAGG--ACAAC-CTTATGTGTATGATACGCCTTTCGCCAGA 169
PBV/Mongoose/KNA/M46/2017        TAGTTCAGAAAGG--ACAAC-CTGAAGTGTATAGCACACCATTTGCTAGA 172
PBV/Mongoose/KNA/M58/2017        ACGTCGTTAAAGG--ACAAC-CTTATGTATATGATACGCCTTTCGCCAGA 169
PBV/Mongoose/KNA/M67/2017        ACGTCGTTAAAGG--ACAAC-CTTATGTATATGATACGCCTTTCGCCAGA 169
PBV/Mongoose/KNA/M72/2017        GTGTTGTCAAAGG--ACAGT-CCGAAGTTTACGACACGCCCTTCGCCAGG 206
PBV/Human/CHN/1-CHN-97/1997      GAGTT-CGATCAG--GCAATACTGACGAATATCGTACTACATTTTTCCGT 180
                                             *   *       *           *  * **

PBV-like/Mongoose/KNA/M17A/2017  ---GACGAGA-AGCCTAGATCATCGATCATGGCGATGTGACAAGCTGTGG 266
PBV/Mongoose/KNA/M17B/2017       ---GGAAGCACAATTCAGAGTGTT--C--TGGATGACTGGCAGGAGGTCC 245
PBV/Mongoose/KNA/M33/2017        ---GGTGAGTCAACTGAGGAACTC--C--TTAGTAAATGGATGGACGTTC 270
PBV/Mongoose/KNA/M45/2017        ---GGCGAATCAACTGCCGAACTC--CTATCGGCGTG-GGATAAG-ACCC 212
PBV/Mongoose/KNA/M46/2017        CACAATGGAGGAACACAGAAGGTT--C--TCAAAGACTGGCTGCC-ACAG 217
PBV/Mongoose/KNA/M58/2017        ---GGCGAATCAACTGCCGAACTC--CTATCGGCGTG-GGATAAG-ACCC 212
PBV/Mongoose/KNA/M67/2017        ---GGCGAATCAACTGCCGAACTC--CTATCGGCGTG-GGATAAG-ACCC 212
PBV/Mongoose/KNA/M72/2017        ---GGCGAATCAACCAAGGAGATG--C--TTGGCAAATGGATGGCTGTGC 249
PBV/Human/CHN/1-CHN-97/1997      ---GGGAGATCATTAGACAGTATC--C--TCAAAGCGTGGAAGCCAACG- 222
                                            *          *      *        *

PBV-like/Mongoose/KNA/M17A/2017  TTGACTCTTCAAATCTGGAAGCTAAGTTGCCAGGTTTGTATAAATTTGAG 316
PBV/Mongoose/KNA/M17B/2017       TTGTATC----AA-TTTCTGACAA-GTGGCCTTCTCTGTGGGAGTTTGAG 289
PBV/Mongoose/KNA/M33/2017        TTGTATC----AA-TCAA-GACGCGGTGGCCATCCTTATGGGAGTTTGAA 314
PBV/Mongoose/KNA/M45/2017        TTGTATC----GA-TTAGCGACAA-GTGGCCCTCGCTGTATGAGTTTGAA 256
PBV/Mongoose/KNA/M46/2017        TTGGATA----AAATTTCTGA-ACAGTGGCCAACACTGCTAGAATTTGAG 262
PBV/Mongoose/KNA/M58/2017        TTGTATC----GA-TTAGCGACAA-GTGGCCCTCGCTGTATGAGTTTGAA 256
PBV/Mongoose/KNA/M67/2017        TTGTATC----GA-TTAGCGACAA-GTGGCCCTCGCTGTATGAGTTTGAA 256
PBV/Mongoose/KNA/M72/2017        TAAAGTC----GA-TTGAC-ACAGCGTGGCCTTCATTGTACAAGTACGAA 293
PBV/Human/CHN/1-CHN-97/1997      TTGGACA----AGATCGAGAACGA-TTGGCCAACGCTTCTGGATTTTGAA 267
                                 *                         * ***     *     * *  **

PBV-like/Mongoose/KNA/M17A/2017  ATGGACATGAAATCAAAAGTCGGTCCACTCTCCGTCCA-AGAACCCTTAC 365
PBV/Mongoose/KNA/M17B/2017       ATGGACTTAGCTAGCAAAGTCGGCCCATTATCAGTTCA-GAAACCTTTGC 338
PBV/Mongoose/KNA/M33/2017        GAGGACCTCTCTAAGAAGGTCGGACCAATGTCAGT-CATGAAACCTTTGA 363
PBV/Mongoose/KNA/M45/2017        ATTGACCTCGCGAAGAAGGTCGGACCTATGTCAGT-CATGGCACCCCTTA 305
PBV/Mongoose/KNA/M46/2017        AAAGATCTTGCGACGAAGGTCGGACCAATGTCTGT-GATGAAACCTCTTA 311
PBV/Mongoose/KNA/M58/2017        ATTGACCTCGCGAAGAAGGTCGGACCTATGTCAGT-CATGGCACCCCTTA 305
PBV/Mongoose/KNA/M67/2017        ATTGACCTCGCGAAGAAGGTCGGACCTATGTCAGT-CATGGCACCCCTTA 305
PBV/Mongoose/KNA/M72/2017        ATTGACCTCGCAAGCAAGGTCGGACCTATGTCAGTGCGTAA-GCCTCTCT 342
PBV/Human/CHN/1-CHN-97/1997      AATGATCTCGCGAAGAAGGTCGGTCCGTTGTCTATTCA-AAAGCCACTCG 316
                                    **  *       ** ***** **  * **  *        **  *
```

Supplementary material S4 (contd.)

```
PBV-like/Mongoose/KNA/M17A/2017  TAAGACGGATTCCCGACGTAATGGACTATTATAACGAT----CCGAAAGG 411
PBV/Mongoose/KNA/M17B/2017       GCGAGAGGATAACTGATATTGCGCATTACTACGAAGATATTCTCCTATCT 388
PBV/Mongoose/KNA/M33/2017        GTCAGAGAATGACTGATATTCGTTCTTACTATGATGGTATTCTCCTTCCA 413
PBV/Mongoose/KNA/M45/2017        GTAAACGCATGCCTGATATAGATTCTTACTACGAAGGTATTCTCTCCCCT 355
PBV/Mongoose/KNA/M46/2017        GCGAACGCATTATGGATATTGATTCTTATTACGACGGTATTCACCAACCA 361
PBV/Mongoose/KNA/M58/2017        GCAAACGCATGTCTGATATAGATTCTTACTACGAAGGTATTCTCTCCCCT 355
PBV/Mongoose/KNA/M67/2017        GTAAACGCATGTCTGATATAGATTCTTACTACGAAGGTATTCTCTCCCCT 355
PBV/Mongoose/KNA/M72/2017        CCGAGCGCATGGAAGACATAGACCATTACTATGACGGTATTCTCCTTCCG 392
PBV/Human/CHN/1-CHN-97/1997      CAGAGCGTTTGGATGACATCGAATCTTACTATGAATCTATCTCCTTAGAT 366
                                       *  *    **  *       ** **  *   *     *

PBV-like/Mongoose/KNA/M17A/2017  TCAGGA--AGTTAGTCCGCGAGCAATCGCATGCG-TTAAGGAAGAATTTA 458
PBV/Mongoose/KNA/M17B/2017       TCAACACCAATAGATCCTGTTGCCATAA-AGCAGACTATTGGTGAATTCA 437
PBV/Mongoose/KNA/M33/2017        TCAGAACCACTCAGTGAGAGGGCGATCGCAGCTG-TGATCTCTGAGTGGC 462
PBV/Mongoose/KNA/M45/2017        TCGAAACCAGTCAGCGACCGAGCAGTAGCGGCCG-TGATCGCTGAGTGGA 404
PBV/Mongoose/KNA/M46/2017        GTCGCACGAATCAACGATACTGCTCTCCAAGCCG-TAGTATCGGAATTCA 410
PBV/Mongoose/KNA/M58/2017        TCGAAACCAGTCAGCGACCGAGCAGTAGCGGCCG-TGATCGCTGAGTGGA 404
PBV/Mongoose/KNA/M67/2017        TCGAAACCAGTCAGCGACCGAGCAGTAGCGGCCG-TGATCGCTGAGTGGA 404
PBV/Mongoose/KNA/M72/2017        TCAACATCTGTGAGCGACAGTGCGATAGCGGCTG-TTGTTCATGAGATGA 441
PBV/Human/CHN/1-CHN-97/1997      TCAGAACCCATTAGTAGATCAGCAATAGCAGCTG-TGATAGCTGAATGGG 415
                                      *    *          **  *       *         **

PBV-like/Mongoose/KNA/M17A/2017  AAGCATTAAGGGGTCTCAGGTTGCGTGATGCTGATGCAACTGTCGCTGAG 508
PBV/Mongoose/KNA/M17B/2017       ACAAAGCAAGAGGGTTGAGAAGAAGATCACAGGCAAGAACAGTAGAAATG 487
PBV/Mongoose/KNA/M33/2017        GTAGTTTCGGAGGACTACGACCCCGCAATCAAGCGGATACCCTTTCGCTC 512
PBV/Mongoose/KNA/M45/2017        GCAGGGTCCGTGGCCTGCACGTGCGTGATCAGAGGAAAACAGTTGAGTTG 454
PBV/Mongoose/KNA/M46/2017        GTCCAATCAGGGGACTGCATCTGCGTAGTCAGCAACGTACAGTCGACTTA 460
PBV/Mongoose/KNA/M58/2017        GCAGGGTCCGTGGCCTGCACGTGCGTGATCAGAGGAAAACAGTTGAGTTG 454
PBV/Mongoose/KNA/M67/2017        GCAGGGTCCGTGGCCTGCACGTGCGTGATCAGAGGAAAACAGTTGAGTTG 454
PBV/Mongoose/KNA/M72/2017        GCAGGTTGCGCGGCCTGCGACTTCGCAGCCAAGATAGGACAGTAGAGTTG 491
PBV/Human/CHN/1-CHN-97/1997      GGGATGCGCGCGGCATCCAATTAAGAGGCCAACGCAAAACAGTGGATAAT 465
                                          * **  *        *             **  *

PBV-like/Mongoose/KNA/M17A/2017  ATGCGCCGCAATACTAATTCGGGCGCTCCTTATTTCACTCGTCGGTCACG 558
PBV/Mongoose/KNA/M17B/2017       ATGAAGAAATCCACCAATTCAGGTAATCCGTTCTTTACAAAACGCAGAAA 537
PBV/Mongoose/KNA/M33/2017        ATGAAGCTGAGCACGAATTCTGGCTCGCCGTACTTTACCAAGAGGCGTAA 562
PBV/Mongoose/KNA/M45/2017        ATGAAGAAATCAACCAATTCCGGCTCTCCGTTCTTCACTAGACGTAAGCG 504
PBV/Mongoose/KNA/M46/2017        ATGAAGAAGTCCACTAACTCTGGGTCACCATACTTCACCAAGAGGCGTAG 510
PBV/Mongoose/KNA/M58/2017        ATGAAGAAGTCAACCAATTCCGGCTCTCCGTTCTTCACTAGACGTAAGCG 504
PBV/Mongoose/KNA/M67/2017        ATGAAGAAGTCAACCAATTCCGGCTCTCCGTTCTTCACTAGACGTAAGCG 504
PBV/Mongoose/KNA/M72/2017        ATGAAGAAGAGCACCAACTCCGGGAGCCCGTACTTCTCAAAGCGTCGATT 541
PBV/Human/CHN/1-CHN-97/1997      ATGAAGAAATCTACTAACTCAGGTTCGCCATTCTTTACTAAACGTAGAGC 515
                                 ***         ** ** ** **    ** *  **  *     *

PBV-like/Mongoose/KNA/M17A/2017  TGTCG-AGTCGGAA---TCCC---TAGAATTGG--------C-------- 585
PBV/Mongoose/KNA/M17B/2017       TGTAGTACTGGATACATTTCCATGTGGTGTTGATTATACACCCAATCCTG 587
PBV/Mongoose/KNA/M33/2017        TGCCATAGACATCACTGTGCCATGTAGTGTCAGAT------TCAACAGTG 606
PBV/Mongoose/KNA/M45/2017        TGTAGTAGACGAAACAATTCCCGCCGAAGTAG---------CACGTCGAG 545
PBV/Mongoose/KNA/M46/2017        AGTTGTAGAGAAAACCGTCCCATGTCG-GTCAAA-------TGGGCAACT 552
PBV/Mongoose/KNA/M58/2017        TGTAGTAGACGAAACAATTCCCGCCGAAGTAG---------CACGTCGAG 545
PBV/Mongoose/KNA/M67/2017        TGTAGTAGACGAAACAATTCCCGCCGAAGTAG---------CACGTCTAG 545
PBV/Mongoose/KNA/M72/2017        AGTCACAGAGCGAACGGTACCGTGCGAAGTCTATC------CGTATCGCG 585
PBV/Human/CHN/1-CHN-97/1997      AGTTGTTGACAAAACTATACC-CGCGAGGTTAGAT---TATCTAGGAGTG 561
                                  *           *   * **        *
```

Supplementary material S4 (contd.)

```
PBV-like/Mongoose/KNA/M17A/2017  -----------TAAG--------------ACTA---------GATCGTTA 601
PBV/Mongoose/KNA/M17B/2017       TATATTATTTGCAGGATGCGTAT-CAGATGTTACCCAACAATAACGATTT 636
PBV/Mongoose/KNA/M33/2017        ----------ATGAAGTA---ATCCAGCAACTAGGCT-C--TGACTTGTG 640
PBV/Mongoose/KNA/M45/2017        ----------GCGAGGTCGTCATGCAAACACTACCAG-C--TGGCATATG 582
PBV/Mongoose/KNA/M46/2017        ----------ACAATTTCTCGAT---GGTGGTGAGAA-----AACCTATG 584
PBV/Mongoose/KNA/M58/2017        ----------GCGAGGTCGTCATGCAAACACTACCAG-C--TGGCATATG 582
PBV/Mongoose/KNA/M67/2017        ----------GCGAGGTCGTCATGCAAACACTACCAG-C--TGGCATATG 582
PBV/Mongoose/KNA/M72/2017        ----------ATGAGATC--CAC-CAGTCACTGAAAGAC--TCGCA-GTG 619
PBV/Human/CHN/1-CHN-97/1997      -----------CAGACT----AAGCAGTTCCTAAATAATTCGGATTGGTA 596
                                                                *                *

PBV-like/Mongoose/KNA/M17A/2017  GCATATC-CAGCAGTTTTAGGGTGGAGAGGACAAGAAGGTGGTATTGATA 650
PBV/Mongoose/KNA/M17B/2017       TAAAGCCTGTGCCGTTATTGGTTGGAGAGGACAGGAAGGTGGACCCGAAG 686
PBV/Mongoose/KNA/M33/2017        GAATGCCTGTGCTGTATTAGGGTGGCGTGGCCAAGAGGGTGGCCCTAACA 690
PBV/Mongoose/KNA/M45/2017        GGATGCGTGTGCTGTTCTAGGTTGGAGAGGTCAGGAAGGCGGTCCCTCCA 632
PBV/Mongoose/KNA/M46/2017        --ATGTTGCAGCTATTCTTGGATGGAGGGGCCAGGAGGGCGGCCCTGCGA 632
PBV/Mongoose/KNA/M58/2017        GGATGCGTGTGCTGTTCTAGGTTGGAGAGGTCAGGAAGGCGGTCCCTCCA 632
PBV/Mongoose/KNA/M67/2017        GGATGCGTGTGCTGTTCTAGGTTGGAGAGGTCAGGAAGGCAGTCCCTCCA 632
PBV/Mongoose/KNA/M72/2017        GAACGCTGCCGCCGTTTTAGGGTGGCGAGGGCAAGAAGGCGGTCCTAAAG 669
PBV/Human/CHN/1-CHN-97/1997      TAGTGC---AGCAGTATTAGGATGGAGAGGACAAGAGGGCGGTCCAACAG 643
                                           **  *  * ** *** * ** ** ** **  *

PBV-like/Mongoose/KNA/M17A/2017  CTGATAACGTGAAACAACGCGTTGTTTGAATGTTTCCTTTCCAAACGAAC 700
PBV/Mongoose/KNA/M17B/2017       ATGAGGATGTTAAACAGAGAGTGGTTTGGATGTTTCCATTCGCTGTTAAT 736
PBV/Mongoose/KNA/M33/2017        AGAAGGATGTAAAGCAGAGGGTTGTTTGGATGTTTCCTTTTGCTGTTAAC 740
PBV/Mongoose/KNA/M45/2017        AGGACGATACGAAACAGCGAGTCGTTTGGATGTTTCCGATGGCTGTTAAC 682
PBV/Mongoose/KNA/M46/2017        CCGGAGATGTTAAACAAAGGGTTGTATGGATGTTTCCCTTTGCAATTAAT 682
PBV/Mongoose/KNA/M58/2017        AGGACGATACGAAACAGCGAGTCGTTTGGATGTTTCCGATGGCTGTTAAC 682
PBV/Mongoose/KNA/M67/2017        AGGACGATACGAAACAGCGAGTCGTTTGGATGTTTCCGATGGCTGTTAAC 682
PBV/Mongoose/KNA/M72/2017        CAAGTGATGTTAAACAGCGTGTGGTTTGGATGTTTCCCTACGCCGTTAAC 719
PBV/Human/CHN/1-CHN-97/1997      ATGAAGACGTTAAACAAAGGGTGGTGTGGATGTTTCCCTTTGGTGTTAAC 693
                                       *    ** **  * ** ** ** ********          **

PBV-like/Mongoose/KNA/M17A/2017  ATCGCGGAATTACAGTTCTACCAGCCCATGATCCGGGCGATTCAAGCACA 750
PBV/Mongoose/KNA/M17B/2017       ATCGAAGAACTCACAGTCTATCAACCACTGATTGAAACATTTCAGAGATT 786
PBV/Mongoose/KNA/M33/2017        ATCAGAGAACTTCAGGTATATCAACCCTTAATTGAAGTTGCACAAAGGGC 790
PBV/Mongoose/KNA/M45/2017        GTACGAGAACTCCAAGTGTATCAGCCCCTCATCGAAGCCGCGCAAAAGTT 732
PBV/Mongoose/KNA/M46/2017        ATTCTCGAGCTCCAAGTCTACCAACCACTCATTGAATCGGCGCAGAGATT 732
PBV/Mongoose/KNA/M58/2017        GTACGAGAACTCCAAGTGTATCAGCCCCTCATCGAAGCCGCGCAAAAGTT 732
PBV/Mongoose/KNA/M67/2017        GTACGAGAACTCCAAGTGTATCAGCCCCTCATCGAAGCCGCGCAAAAGTT 732
PBV/Mongoose/KNA/M72/2017        ATCGCAGAATTGCAGCTGTATCAACCATTCATTGAGGCCGCACAAAGGCA 769
PBV/Human/CHN/1-CHN-97/1997      GTGAAGGAACTTCAATTCTACCAACCAGCAATCGAAATCGCACAGAAG-- 741
                                  *    **  *     * ** ** **    **          **

PBV-like/Mongoose/KNA/M17A/2017  TAAAC--TCATCCCTGCCTATTTGGATCATACA-GCAGTGGATGCTCGGA 797
PBV/Mongoose/KNA/M17B/2017       CAATC--TGGTTCCAGCTTGGGTGAG-TATGGAAAGTGTTGATATGGAAG 833
PBV/Mongoose/KNA/M33/2017        AAACT--TGGTTCCTGCATGGGTTAG-CATGGAATCGGTTGATAAGGAAA 837
PBV/Mongoose/KNA/M45/2017        CGATG--TGGTCCCTGCGTGGGTTAG-CATGGATCAGGTTGATTTGGCCG 779
PBV/Mongoose/KNA/M46/2017        CAATT--TGGTTCCATCGTGGGTTTC-GATGGAATCCGTGGATCAACGTA 779
PBV/Mongoose/KNA/M58/2017        CGATG--TGGTCCCTGCGTGGGTTAG-CATGGATCAGGTTGATTTGGCCG 779
PBV/Mongoose/KNA/M67/2017        CGATG--TGGTCCCTGCGTGGGTTAG-CATGGATCAGGTTGATTTGGCCG 779
PBV/Mongoose/KNA/M72/2017        CAATG--TCGTCCCAGCTTGGGTTGG-CTTCGACGAGGTTGATAAACGCA 816
PBV/Human/CHN/1-CHN-97/1997      CGATGGATAACTCCTGCATGGATTGG-CATGGAGGCAGTAGATCAGCGAA 790
                                   *    *    **  * *   *      *  *    ** ***
```

Supplementary material S4 (contd.)

```
PBV-like/Mongoose/KNA/M17A/2017  TAACGAATCTATTTGACACCAAACCTACGGGTGATCTTGTAGTTGAAACC 847
PBV/Mongoose/KNA/M17B/2017       TCACTCGCTTATTTGATACTAAGAATAGTGACGACCTCGTTGTGTGCACG 883
PBV/Mongoose/KNA/M33/2017        TTACACGATTATTTGATACTAAGAGTGATAATGACCTGATAGTCTGTACC 887
PBV/Mongoose/KNA/M45/2017        TGACACGCCTGTTCGATACTAAGGGCGCGAACGACCTCGTAGTATGCACC 829
PBV/Mongoose/KNA/M46/2017        TTACTCGTATGTTTGATACCAAGGCAGACGGTGATTTGGTAGTATGCACC 829
PBV/Mongoose/KNA/M58/2017        TCACGCGCCTGTTCGATACTAAGGGCGCAAACGACCTCGTAGTATGCACC 829
PBV/Mongoose/KNA/M67/2017        TGACGCGCCTGTTCGATACTAAGGGCGCGAACGACCTCGTAGTATGCACC 829
PBV/Mongoose/KNA/M72/2017        TTACAGCCATGTTCGACACAAAGGCTGCTAATGATCTAGTCGTATGTACC 866
PBV/Human/CHN/1-CHN-97/1997      TCACTAAGCTGTTCGATACTAAGGCTAAGTCAGAGTTGGTAATCTGTACA 840
                                 * **     * ** ** ** **          **  *  *  *    **

PBV-like/Mongoose/KNA/M17A/2017  GATTTCACCAAGTTCGACCAGTCGTTCTGAACACCAT-TACAACATGTAG 896
PBV/Mongoose/KNA/M17B/2017       GACTTCACAAAATTCGACCAG-CATTTCAATGTAGATCTACAGAATGCAG 932
PBV/Mongoose/KNA/M33/2017        GACTTCTCAAAATTCGACCAG-CACTTTGGTCCTGATATGCAGGATGCTG 936
PBV/Mongoose/KNA/M45/2017        GATTTCACCAAGTTTGACCAA-CACTTCAATCATTCACTTCAAGTTGCTG 878
PBV/Mongoose/KNA/M46/2017        GATTTTGAAAAGTTCGACCAG-CACTTCAACTCCGCTCTGCAACTAGCGG 878
PBV/Mongoose/KNA/M58/2017        GATTTCACCAAGTTTGACCAA-CACTTCAATCATTCACTTCAAGTTGCTG 878
PBV/Mongoose/KNA/M67/2017        GATTTCACCAAGTTTGACCAA-CACTTCAATCATTCACTTCAAGTTGCTG 878
PBV/Mongoose/KNA/M72/2017        GACTTCAGCAAGTTCGACCAA-CATTTTAACGCCAAATTGCAAGATGCTG 915
PBV/Human/CHN/1-CHN-97/1997      GACTTCAGCAAGTTCGACCAA-CATTTTAACAAGGATATGCAGGATGCAG 889
                                 ** **    ** ** *****  *  *            * **   *   *

PBV-like/Mongoose/KNA/M17A/2017  CGCGTGCTGT-----GTACG---CCGACTGAGGTCCACAAC-------TT 931
PBV/Mongoose/KNA/M17B/2017       CGAAAACCAT----ACTGCA--ATGGATCATGTCACGCGAAAGAAATGTA 976
PBV/Mongoose/KNA/M33/2017        CAAGAGCCAT----ATTGCA--GAAAATCCTGTCACC--AC---AAGCTT 975
PBV/Mongoose/KNA/M45/2017        CCCTATCCAT----TCTGGA--AGCAATCTTTACACCCGACCGGGAGTGG 922
PBV/Mongoose/KNA/M46/2017        CAGAACAAAT----CCTACG--CAGGATACTCGCTCCGGATGCGGACAGT 922
PBV/Mongoose/KNA/M58/2017        CCCTATCCAT----TCTGGA--AGCAATCTTTACACCCGACCGGGAGTGG 922
PBV/Mongoose/KNA/M67/2017        CCCTATCCAT----TCTGGA--AGCAATCTTTACACCCGACCGGGAGTGG 922
PBV/Mongoose/KNA/M72/2017        CTCTTCAAGT----GCTTCG--AGCAATAGTAACACCCGGCGTGACGTCC 959
PBV/Human/CHN/1-CHN-97/1997      CTAGGACCATTATCGCTGCATTACTGAATGGATCATCTGA----AAGC-- 933
                                 *        *      *         *

PBV-like/Mongoose/KNA/M17A/2017  GATAAGTGAATTAGAGAAGTATTCCCGGTAAAATATAATATTCCCATGG- 980
PBV/Mongoose/KNA/M17B/2017       GATGACTGGTTTGAAAACGTGTTCGGAATCAAATATATGATTCCCTTA-- 1024
PBV/Mongoose/KNA/M33/2017        CA-GACTGGTTGAGAGAAGTATTTCCCATTAAGTATATGATCCCGTTGGC 1024
PBV/Mongoose/KNA/M45/2017        CAGGAATGGTCCCGTGAAATCTATCCCCTGAAATATATGATTCCTTTAGC 972
PBV/Mongoose/KNA/M46/2017        CTAAACTGGTTACGACAAATTTTCCCCATTAAGTTCAATATCCCATTAGC 972
PBV/Mongoose/KNA/M58/2017        CAGGAATGGTCCCGTGAAATCTATTCCCTGAAATATATGATTCCTTTAGC 972
PBV/Mongoose/KNA/M67/2017        CAGGAATGGTCCCGTGAAATCTATTCCCTGAAATATATGATTCCTTTAGC 972
PBV/Mongoose/KNA/M72/2017        AAAGAATGGTTCGACAAAGTGTACCCCATCAAGTACATGATACCTTTAGC 1009
PBV/Human/CHN/1-CHN-97/1997      CGTGATTGGTTGGACAACGTTTTCCCAGTGAAGTATGCGATACCTCTAGC 983
                                     * **        * * *      * ** *     ** **  *

PBV-like/Mongoose/KNA/M17A/2017  -TATTAT-CGAATAATTTGATTTC-----------CACCGGACCACACGG 1017
PBV/Mongoose/KNA/M17B/2017       CTGTGGGAAGCACAAGCTGATATTGTATCAGTTTTCCACGGATTTCACGG 1074
PBV/Mongoose/KNA/M33/2017        TATCCAGGATGATAAAATACTTTA--------------TGGACGTCATGG 1060
PBV/Mongoose/KNA/M45/2017        TTATGACTACGGTAAGCTGAGAAC--------------AGGGCCGCACGG 1008
PBV/Mongoose/KNA/M46/2017        TGTTGATATGAACACCGTGTTTAC--------------AGGTGCGCACGG 1008
PBV/Mongoose/KNA/M58/2017        TTATGACTACGGTAAGCTGAGAAC--------------AGGGCCGCACGG 1008
PBV/Mongoose/KNA/M67/2017        TTATGACTACGGTAAGCTGAGAAC--------------AGGGCCGCACGG 1008
PBV/Mongoose/KNA/M72/2017        ATATGATGTGGGTAGGGTTAGATT--------------TGGGAAACATGG 1045
PBV/Human/CHN/1-CHN-97/1997      TATGGATATGGGAGTAGTAAAAAC--------------TGGTTTTCACGG 1019
                                                  *                     **    ** **
```

Supplementary material S4 (contd.)

```
PBV-like/Mongoose/KNA/M17A/2017  CATGGCGTCGGGATCAGGGGGTACTAATGCTGATGAGTCGATTGCACACA 1067
PBV/Mongoose/KNA/M17B/2017       AATGGCTTCTGGTTCGGGAGGTACGAACGTAGATGAGACTTTAGCTCATC 1124
PBV/Mongoose/KNA/M33/2017        CATGGGTTCAGGCAGTGGAGGAACAAACGCCGACGAAACGTTAGCTCACC 1110
PBV/Mongoose/KNA/M45/2017        AATGGGCTCAGGTTCTGGTGGAACCAATGCTGATGAGACTTTAGCTCATC 1058
PBV/Mongoose/KNA/M46/2017        TATGGGAAGTGGTTCCGGGGGTACCAATGCTGACGAAACGTTAGCACACA 1058
PBV/Mongoose/KNA/M58/2017        AATGGGCTCAGGTTCTGGTGGAACCAATGCTGATGAGACTTTAGCTCATC 1058
PBV/Mongoose/KNA/M67/2017        AATGGGCTCAGGTTCTGGTGGAACCAATGCTGATGAGACTTTAGCTCATC 1058
PBV/Mongoose/KNA/M72/2017        AATGGGCTCTGGTTCGGGAGGAACGAACTGTGACGAAACTTTAGCCCATC 1095
PBV/Human/CHN/1-CHN-97/1997      TATGGGTAGTGGTTCAGGTGGAACCAACTTCGATGAAACCCTGACACATC 1069
                                  ****     **    ** ** ** **    ** **  *  *  * **

PBV-like/Mongoose/KNA/M17A/2017  GGGCGTTACAGCATGAAGCTGC--TTTAGCGTCGGGTCAGACTCTCCACC 1115
PBV/Mongoose/KNA/M17B/2017       GAGCCCTTCAACGTGAGGCGGC-CATAAAAAGTAAACAAATAT-TAAACC 1172
PBV/Mongoose/KNA/M33/2017        GTGCCCTCCAATATGAAGCTGCCCAG-CAAATGGGTGCCAGAT-TAAACC 1158
PBV/Mongoose/KNA/M45/2017        GAGCTCTGCAATATGAGGCCGC-CATTCGTGCGAAGACTCGTT-TAAACC 1106
PBV/Mongoose/KNA/M46/2017        GAGCTCTTCAATATGAGGCCGC-CATCAAAAACAACTCCCGAT-TAAACC 1106
PBV/Mongoose/KNA/M58/2017        GAGCTCTGCAATATGAGGCCGC-CATTCGTGCGAAGACTCGTT-TAAACC 1106
PBV/Mongoose/KNA/M67/2017        GAGCTCTGCAATATGAGGCCGC-CATTCGTGCGAAGACTCGTT-TAAACC 1106
PBV/Mongoose/KNA/M72/2017        GTGCACTGCAATATGAGGCAGCTCAAACTAGCGGGGTAAAGCT-TAA-CC 1143
PBV/Human/CHN/1-CHN-97/1997      GAGCTTTACAGTATGAAGCAGCCCAG-AAAGCTAAATCGGTGC-TCAATC 1117
                                 * **  * **   *** ** **                      *    *

PBV-like/Mongoose/KNA/M17A/2017  CATCCTCAATGGCATATGGGGACGATGGTATCTTAACGTTCAAGGGTATC 1165
PBV/Mongoose/KNA/M17B/2017       CTCATTCAATGTGTCTTGGCGATGATGGGATACTAAGTTATCCCGGAATA 1222
PBV/Mongoose/KNA/M33/2017        CAAGTTCAATGTGTTTAGGCGATGATGGAATCTTGAGCTATCCAGGCATC 1208
PBV/Mongoose/KNA/M45/2017        CGAATTCACAGTGTCTTGGCGATGATGGGATACTAAGTTATCCTGGAATC 1156
PBV/Mongoose/KNA/M46/2017        CTAATTCACAGTGCTTGGGCGATGATGGTGTATTAACATACCCAGGCATT 1156
PBV/Mongoose/KNA/M58/2017        CGAATTCACAGTGTCTTGGCGATGACGGGATACTAAGTTATCCTGGAATC 1156
PBV/Mongoose/KNA/M67/2017        CGAATTCACAGTGTCTTGGCGATGACGGGATACTAAGTTATCCTGGAATC 1156
PBV/Mongoose/KNA/M72/2017        CAAATTCACAGTGTCTTGGCGATGACGGGGTGCTAACCTACCCTGGCATC 1193
PBV/Human/CHN/1-CHN-97/1997      CAAATTCGCAGTGTCTAGGTGATGACGGCATTCTTTCCTATCCTGGAATT 1167
                                 *    **   *      ** ** ** **  *  *    *     ** **

PBV-like/Mongoose/KNA/M17A/2017  ACCGTTGATAAAGTGGTAGCACAATATACTAAGCATGGAGTGACCATGAA 1215
PBV/Mongoose/KNA/M17B/2017       ACAGTGAATGAAGTAGTGGATGCATACCAGTCTCACGGTCAAGAGTGCAA 1272
PBV/Mongoose/KNA/M33/2017        ACAGTGGACGATGTAGTTAAAGCGTATAGTAGGCACGGGCTTGAAATGAA 1258
PBV/Mongoose/KNA/M45/2017        ACTGTGGAAGATGTAGTGCAATCGTATACTGAACATGGTCTCGAGATGAA 1206
PBV/Mongoose/KNA/M46/2017        ACTGCGGAGGATGTAATGCAATCGTACAAAAGACATGGCCTCAAAATGAA 1206
PBV/Mongoose/KNA/M58/2017        ACTGTGGAAGATGTAGTGCAATCGTATACTGAACATGGTCTCGAGATGAA 1206
PBV/Mongoose/KNA/M67/2017        ACTGTGGAAGATGTAGTGCAATCGTATACTGAACATGGTCTCGAGATGAA 1206
PBV/Mongoose/KNA/M72/2017        ACTGTGGAGGATGTAGTCAAAGCGTACACGTCACATGGGGTTGAGATGAA 1243
PBV/Human/CHN/1-CHN-97/1997      ACTGTGGAGGATGTAACACGTTCGTATTCTGCGCATGGTCAGGAAATGAA 1217
                                 ** *   *  * **          **       ** **          **

PBV-like/Mongoose/KNA/M17A/2017  CGAGAGT--AAGCAGGGAGCAAGCACTACCGAGTGCGTATTCCTACGCCG 1263
PBV/Mongoose/KNA/M17B/2017       TTTAAGC--AAACAGTATGCATCAACCCAAGATTGCGTATATCTCAGAAG 1320
PBV/Mongoose/KNA/M33/2017        TCAGGAC--AAGCAATACGCTTCAACCCAAGATTGCGTGTACTTGAGAAG 1306
PBV/Mongoose/KNA/M45/2017        T--GAATCGAAGCAGTACGCGAGCACCCATGACTGCACATACTTAAGAAG 1254
PBV/Mongoose/KNA/M46/2017        CGCTAGT--AAACAGTACGAGAGCACACAGGACTGCACATACTTGCGAAG 1254
PBV/Mongoose/KNA/M58/2017        T--GAATCGAAGCAGTACGCGAGCACCCATGACTGCACATACTTAAGAAG 1254
PBV/Mongoose/KNA/M67/2017        T--GAATCGAAGCAGTACGCGAGCACCCATGACTGCACATACTTAAGAAG 1254
PBV/Mongoose/KNA/M72/2017        TCTGGGT--AAACAGTACGCCTCGACTCAAGATTGCATATATCTTAGGCG 1291
PBV/Human/CHN/1-CHN-97/1997      TATTGAT--AAGCAGTATGCGAGTGCACATGACTGCACATATCTTCGTCG 1265
                                          ** **    *      *    ** ***   *   *  *  *
```

Supplementary material S4 (contd.)

```
PBV-like/Mongoose/KNA/M17A/2017  TTGACACTCTATGTCCTATCGTAAGAACGGGATTATG-GTAGGTGTTTAT 1312
PBV/Mongoose/KNA/M17B/2017       ATGGCATCATAAAGATTATCGGGATAATGGGGT-ATGCGTCGGGGTCTAC 1369
PBV/Mongoose/KNA/M33/2017        GTGGCACCATAAAGATTACCGAATACGAGGAGT-ATGCGCAGGAGTGTAC 1355
PBV/Mongoose/KNA/M45/2017        GTGGTATCACCGCGAGTATAGGATAAAGGGTGT-ATGTGCAGGAGTATAC 1303
PBV/Mongoose/KNA/M46/2017        ATGGCATCATAAAGATTATCGCATGGACGGTAT-GTGTGTAGGCGTATAC 1303
PBV/Mongoose/KNA/M58/2017        GTGGTATCACCGCGAGTATAGGATAAAGGGTGT-ATGTGCAGGAGTATAC 1303
PBV/Mongoose/KNA/M67/2017        GTGGTACCACCGCGAGTATAGGATAAAGGGTGT-ATGTGCAGGAGTATAC 1303
PBV/Mongoose/KNA/M72/2017        GTGGCACCACAAAGACTACCGCGTGGACGGGGT-ATGTGCAGGGGTATAC 1340
PBV/Human/CHN/1-CHN-97/1997      GTGGCACCACGACGACTACAGAGTAGACGGTGT-ATGTGTAGGTGTTTAT 1314
                                  **  *          **  *       **  *  ** *  ** ** **

PBV-like/Mongoose/KNA/M17A/2017  TCGACGTTCCGGGCTTTAGGACGTTTGATGGGTCAAGAACGGTACTATGA 1362
PBV/Mongoose/KNA/M17B/2017       TCGACCTGTCGAGCGCTGGGTAGATTGAGATACCTCGAAAGAT-TTATGG 1418
PBV/Mongoose/KNA/M33/2017        GCTACTACCCGGGCTTTGGGTAGGTTAAGGTACCTCGAGCGAT-TTATGG 1404
PBV/Mongoose/KNA/M45/2017        TCAACTAACCGGGCCTTAGGTAGATTGAGATACTTAGAGCGCTACTATGA 1353
PBV/Mongoose/KNA/M46/2017        TCCACCTGCCGGGCTTTAGGTAGGTTATGCGAGCAAGAGAGATATTACGA 1353
PBV/Mongoose/KNA/M58/2017        TCAACTAACCGGGCCTTAGGTAGATTGAGATACTTAGAACGCTACTATGA 1353
PBV/Mongoose/KNA/M67/2017        TCAACTAACCGGGCCTTAGGTAGATTGAGATACTTAGAACGCTACTATGA 1353
PBV/Mongoose/KNA/M72/2017        AGCACCTGTCGGGCTTTAGGCAGGTTGCGGTATCTCGAACGCTATCAGAA 1390
PBV/Human/CHN/1-CHN-97/1997      GCAACCTACAGAGCGTTAGGTAGGCTGTGTGAACAGGAACGCTATTATGA 1364
                                    **     * **  * **  *  *          **  * *   *

PBV-like/Mongoose/KNA/M17A/2017  -CCCTAAACAATGAGGACCAGAGATGGTTATAATGAGGGCGTTGTCGATA 1411
PBV/Mongoose/KNA/M17B/2017       ATCCAGAGTATTGGAGCCCGCAGATGGTAGCTTTACGCCAATTATCAATA 1468
PBV/Mongoose/KNA/M33/2017        ACCCTGACAACTGGGGACCAAAGATGGTTGCTCTACGACAACTATCAATC 1454
PBV/Mongoose/KNA/M45/2017        -CCCAGAGGTGTGGGGTCCAAAGATGGTTGCCCTCAGACAACTATCTATA 1402
PBV/Mongoose/KNA/M46/2017        -TCCGGAAATATGGTCAAAAGAGATGGTTGCGCTGCGTCAACTATCTATA 1402
PBV/Mongoose/KNA/M58/2017        -CCCAGAGGTGTGGGGTCCAAAGATGGTTGCCCTCAGACAACTATCTATA 1402
PBV/Mongoose/KNA/M67/2017        -CCCAGAGGTGTGGGGTCCAAAGATGGTTGCCCTCAGACAACTATCTATA 1402
PBV/Mongoose/KNA/M72/2017        TCCCAAATAT-TGGGATGCAAAGGCTGTAGCTCTACGTCAGCTAAGCATA 1439
PBV/Human/CHN/1-CHN-97/1997      -TCCAGAAGTATGGGGGCCTAAGATGGTAGCTCTACGGCAGCTTTCAATT 1413
                                   **  *    **        **   **     *  *     *    **

PBV-like/Mongoose/KNA/M17A/2017  CTAGAAAATTGCAACCAACATCCTCTCTTCGAAGAGTTTATTGAATTTGT 1461
PBV/Mongoose/KNA/M17B/2017       ATTGAGAATTGTAAGTACCATCCTCTGCGTGACGAGTTTGCGGAGTTTTG 1518
PBV/Mongoose/KNA/M33/2017        ATTGAGAATGTGAAGTACCATCCAATGAGGGCTGAATTCGTAGAATTTTG 1504
PBV/Mongoose/KNA/M45/2017        TTAGAGAATGTGAAGAACCATCCTCTACGTGATCAATTTGCTCAGTTTTG 1452
PBV/Mongoose/KNA/M46/2017        ATGGAGAATGTGAAGTATCACCCATTACGTGCCGAATTCGCAGACTTTTG 1452
PBV/Mongoose/KNA/M58/2017        TTAGAGAATGTGAAGAACCATCCTCTACGTGATCAATTTGCTCAGTTTTG 1452
PBV/Mongoose/KNA/M67/2017        TTAGAGAATGTGAAGAACCATCCTCTACGTGATCAATTTGCTCAGTTTTG 1452
PBV/Mongoose/KNA/M72/2017        CTTGAGAATGTGAAGTACCATCCTCTCAAGGAACAGTTTGTGGAATTTTG 1489
PBV/Human/CHN/1-CHN-97/1997      ATTGAGAACTGTAAGTACCATCCTCTCCGTGAGGAGTTCGTTAACTTCTG 1463
                                  * ** **    **  * ** **  *    *   * **     * **

PBV-like/Mongoose/KNA/M17A/2017  CATCAAAGGGGATAAGTACGGATTGGGTACTAAACTCCCAGGCTTTTTGG 1511
PBV/Mongoose/KNA/M17B/2017       CATGAAGAGGGATAAATATCGATTGGGTATTGATATCCCAGGATTCTTAG 1568
PBV/Mongoose/KNA/M33/2017        CATGAAAAGGGATAAATATAGACTCGGACTAGATATCCCAGGCTTTCTTG 1554
PBV/Mongoose/KNA/M45/2017        CATGAAAAGGGATAAATATCGCCTTGGTATTGATATCCCAGGTTTCCTAG 1502
PBV/Mongoose/KNA/M46/2017        CATGAAAAGGGATATGTATAGGCTAGGGTTAGATATCCCAGGATTCTTAG 1502
PBV/Mongoose/KNA/M58/2017        CATGAAAAGGGATAAATATCGCCTTGGTATTGATATCCCAGGTTTCCTAG 1502
PBV/Mongoose/KNA/M67/2017        CATGAAAAGGGATAAATATCGCCTTGGTATCGATATCCCAGGTTTCCTAG 1502
PBV/Mongoose/KNA/M72/2017        CATGGTAAGGGATAGATATCGATTAGGACTCGATATTCCCAATTTTCTAG 1539
PBV/Human/CHN/1-CHN-97/1997      CATGAAAGGGGATAAATACAGACTCGGACTGGACATTCCAGGTTTCCTTG 1513
                                 ***     ******  **  *  * **     *  * **    **  * *
```

Supplementary material S4 (contd.)

```
PBV-like/Mongoose/KNA/M17A/2017  ATAAT-TTACAGGCAATG----TACGCCAAAGCCAGAGATGTGATGCCCG 1556
PBV/Mongoose/KNA/M17B/2017       ATAAAATCGCGACTTATG---CTAAA--GAATCTATCGATCACATGCCAG 1613
PBV/Mongoose/KNA/M33/2017        ACGGAATAGAATCTGAAG---CTAAG--AAAGCTATCGATCTAATGCCAG 1599
PBV/Mongoose/KNA/M45/2017        ACGATATTGAGGGTGAGG---CTAGG--AAAGCTATCAACTACATGCCCG 1547
PBV/Mongoose/KNA/M46/2017        ATAATATTGACCGTATCG---CTAAG--GAATCTATCGAAGTCATGCCGG 1547
PBV/Mongoose/KNA/M58/2017        ACGACATTGAGGGTGAGG---CTAGG--AAAGCTATCAACTACATGCCCG 1547
PBV/Mongoose/KNA/M67/2017        ACGACATTGAGGGTGAGG---CTAGG--AAAGCTATCAACTACATGCCCG 1547
PBV/Mongoose/KNA/M72/2017        ACAAT-TTGAGAGAGATTGCTCTAGA--GAAG--ATCGAAGACATGCCAG 1584
PBV/Human/CHN/1-CHN-97/1997      ATAATATCGAAAAATTAG---CAATG--GAAGCTACGGATCTCATGCCAG 1558
                                 *     *                *     **   *   *    ***** *

PBV-like/Mongoose/KNA/M17A/2017  ATTATGGTGGCTTCGTTAATGATAAT-----------ATGAAGCGC--AA 1593
PBV/Mongoose/KNA/M17B/2017       ATTTCTTAGGTTATACCAA-GACTTTACAGGG----GTTCAAGCCC--AC 1656
PBV/Mongoose/KNA/M33/2017        ACTTCCTTGGTTATACCAA-GACGCTGCAGAGCGTTGATGATGTC---GG 1645
PBV/Mongoose/KNA/M45/2017        ACTTCTTGGGCTATACCAA-AACGCTGCAGGCAGATGCGGGAGGT---AT 1593
PBV/Mongoose/KNA/M46/2017        ACTTTCTTGGTTATACCAA-GACCATGATGAAG---AATCAAGAC---GT 1590
PBV/Mongoose/KNA/M58/2017        ACTTCTTGGGCTATACCAA-AACACTGCAGGCAGATGCAGGAGGT---AT 1593
PBV/Mongoose/KNA/M67/2017        ACTTCTTGGGCTATACCAA-AACACTGCAGGCAGATGCAGGAGGT---AT 1593
PBV/Mongoose/KNA/M72/2017        ACTTCTTAGGTTACACCAG-GCAACTCCAATC---TAAAGGAGACCCAGC 1630
PBV/Human/CHN/1-CHN-97/1997      ACTTTCTAGGATATACCAA-GTCACT--AGG----TACAAGTGGCG--AT 1599
                                 * *     ** *     *       *                *

PBV-like/Mongoose/KNA/M17A/2017  AGGAATCCTTTCA---TGGAAGGTAGTCCAATACCTAATTAATC--GGTA 1638
PBV/Mongoose/KNA/M17B/2017       TGGTATATCCGAA---TGGTGGATAGTAAAATATCTGA--AATCGCATCG 1701
PBV/Mongoose/KNA/M33/2017        CGGTATA-TCCAAT--TGGTGGATAGTTAAATATTTGA--AGTCAAAGTA 1690
PBV/Mongoose/KNA/M45/2017        TGGTATA-TCGAAA--TGGTGGATAGTCAACTATCTGA--AGTCCAGGTA 1638
PBV/Mongoose/KNA/M46/2017        AGGCTTG-TC--ATCTTGGTGGATTGTTAATTATCTAA--AGTCTAAGTA 1635
PBV/Mongoose/KNA/M58/2017        TGGTATA-TCGAAA--TGGTGGATAGTCAACTATCTGA--AGTCCAGGTA 1638
PBV/Mongoose/KNA/M67/2017        TGGTATA-TCGAAA--TGGTGGATAGTCAACTATCTGA--AGTCCAGGTA 1638
PBV/Mongoose/KNA/M72/2017        TGGTGGAATCGAAAATTGGTGGGTAGTTAAATACCTGA--AGTCAAAGTA 1678
PBV/Human/CHN/1-CHN-97/1997      AGGGGTA-TCGGACAATGGTGGGTAGTTAACTACCTAA--AGTCTTTACG 1646
                                  **         *   ***  * * **  * **  * *  * **

PBV-like/Mongoose/KNA/M17A/2017  AG-----CGAGATGGTGCAGTAAACCATT--------------------- 1662
PBV/Mongoose/KNA/M17B/2017       ATAAAATCGAGATG------------------------------------ 1715
PBV/Mongoose/KNA/M33/2017        AGGA--TTGAGAT------------------------------------- 1701
PBV/Mongoose/KNA/M45/2017        ACGG--TTGAGATGG----------------------------------- 1651
PBV/Mongoose/KNA/M46/2017        AAT---TCGAGATGGTGC-------------------------------- 1650
PBV/Mongoose/KNA/M58/2017        ACGG--TTGAGATGGTGCAGTAAACCATTAGATCATGAGCATGATCTAAC 1686
PBV/Mongoose/KNA/M67/2017        ACAG--TTGAGATGGTGC-------------------------------- 1654
PBV/Mongoose/KNA/M72/2017        ACT---TCGAGATGGT---------------------------------- 1691
PBV/Human/CHN/1-CHN-97/1997      CTAAA-TCCGGATGGTGCAGCAAACCATTAACTCCTCACGGAGTTAACTG 1695
                                           ***

PBV-like/Mongoose/KNA/M17A/2017  ---
PBV/Mongoose/KNA/M17B/2017       ---
PBV/Mongoose/KNA/M33/2017        ---
PBV/Mongoose/KNA/M45/2017        ---
PBV/Mongoose/KNA/M46/2017        ---
PBV/Mongoose/KNA/M58/2017        TGC 1689
PBV/Mongoose/KNA/M67/2017        ---
PBV/Mongoose/KNA/M72/2017        ---
PBV/Human/CHN/1-CHN-97/1997      C-- 1696
```
