# Supplementary material for: Detection and Molecular Characterization of Picobirnaviruses (PBVs) in the Mongoose: Identification of a Novel PBV Using an Alternative Genetic Code
Source: Viruses. 2020 Jan 15;12(1):99. doi: 10.3390/v12010099 (PMC7019992; doi:10.3390/v12010099)
Supplement: Supplementary file 1 [file viruses-12-00099-s001.zip › Supplementary material S5.pdf]

Supplementary material S5. The three translation frames (5’- 3’) of the novel mongoose PBV-like sequence (designated as M17A) using the standard genetic code. The translation frames were generated by the Translate tool-ExPASY software (https://web.expasy.org/translate/, accessed 16th October 2019). The putative peptides and corresponding coding sequences is highlighted with light pink and yellow, respectively.

5'3' Frame 1

```
gta aat ttt caa aat ttt cta ttt cta tga aat ttt aaa ttg cgt gat tta ctg aaa acg
 V   N   F   Q   N   F   L   F   L   -   N   F   K   L   R   D   L   L   K   T
ctt aca gtg gca gat aac tgt ata cat ttc agg agg tta gtt tct tgt gat taa gaa gct
 L   T   V   A   D   N   C   I   H   F   R   R   L   V   S   C   D   -   E   A
aaa ctt gaa gga cta ttt cat cct tcc caa tca ggg ttt aca gtc tta ttt aca caa gac
 K   L   E   G   L   F   H   P   S   Q   S   G   F   T   V   L   F   T   Q   D
gga ggt agg tag cga cct tga cat tcg tac tcc gtt ttg gga cga gaa gcc tag atc atc
 G   G   R   -   R   P   -   H   S   Y   S   V   L   G   R   E   A   -   I   I
gat cat ggc gat gtg aca agc tgt ggt tga ctc ttc aaa tct gga agc taa gtt gcc agg
 D   H   G   D   V   T   S   C   G   -   L   F   K   S   G   S   -   V   A   R
ttt gta taa att tga gat gga cat gaa atc aaa agt cgg tcc act ctc cgt cca aga acc
 F   V   -   I   -   D   G   H   E   I   K   S   R   S   T   L   R   P   R   T
ctt act aag acg gat tcc cga cgt aat gga cta tta taa cga tcc gaa agg tca gga agt
 L   T   K   T   D   S   R   R   N   G   L   L   -   R   S   E   R   S   G   S
tag tcc gcg agc aat cgc atg cgt taa gga aga att taa agc att aag ggg tct cag gtt
 -   S   A   S   N   R   M   R   -   G   R   I   -   S   I   K   G   S   Q   V
gcg tga tgc tga tgc aac tgt cgc tga gat gcg ccg caa tac taa ttc ggg cgc tcc tta
 A   -   C   -   C   N   C   R   -   D   A   P   Q   Y   -   F   G   R   S   L
ttt cac tcg tcg gtc acg tgt cga gtc gga atc cct aga att ggc taa gac tag atc gtt
 F   H   S   S   V   T   C   R   V   G   I   P   R   I   G   -   D   -   I   V
agc ata tcc agc agt ttt agg gtg gag agg aca aga agg tgg tat tga tac tga taa cgt
 S   I   S   S   S   F   R   V   E   R   T   R   R   W   Y   -   Y   -   -   R
gaa aca acg cgt tgt ttg aat gtt tcc ttt cca aac gaa cat cgc gga att aca gtt cta
 E   T   T   R   C   L   N   V   S   F   P   N   E   H   R   G   I   T   V   L
cca gcc cat gat ccg ggc gat tca agc aca taa act cat ccc tgc cta ttt gga tca tac
 P   A   H   D   P   G   D   S   S   T   -   T   H   P   C   L   F   G   S   Y
agc agt gga tgc tcg gat aac gaa tct att tga cac caa acc tac ggg tga tct tgt agt
 S   S   G   C   S   D   N   E   S   I   -   H   Q   T   Y   G   -   S   C   S
tga aac cga ttt cac caa gtt cga cca gtc gtt ctg aac acc att aca aca tgt agc gcg
 -   N   R   F   H   Q   V   R   P   V   V   L   N   T   I   T   T   C   S   A
tgc tgt gta cgc cga ctg agg tcc aca act tga taa gtg aat tag aga agt att ccc ggt
 C   C   V   R   R   L   R   S   T   T   -   -   V   N   -   R   S   I   P   G
aaa ata taa tat tcc cat ggt att atc gaa taa ttt gat ttc cac cgg acc aca cgg cat
 K   I   -   Y   S   H   G   I   I   E   -   F   D   F   H   R   T   T   R   H
ggc gtc ggg atc agg ggg tac taa tgc tga tga gtc gat tgc aca cag ggc gtt aca gca
 G   V   G   I   R   G   Y   -   C   -   -   V   D   C   T   Q   G   V   T   A
tga agc tgc ttt agc gtc ggg tca gac tct cca ccc atc ctc aat ggc ata tgg gga cga
 -   S   C   F   S   V   G   S   D   S   P   P   I   L   N   G   I   W   G   R
tgg tat ctt aac gtt caa ggg tat cac cgt tga taa agt ggt agc aca ata tac taa gca
 W   Y   L   N   V   Q   G   Y   H   R   -   -   S   G   S   T   I   Y   -   A
tgg agt gac cat gaa cga gag taa gca ggg agc aag cac tac cga gtg cgt att cct acg
 W   S   D   H   E   R   E   -   A   G   S   K   H   Y   R   V   R   I   P   T
ccg ttg aca ctc tat gtc cta tcg taa gaa cgg gat tat ggt agg tgt tta ttc gac gtt
 P   L   T   L   Y   V   L   S   -   E   R   D   Y   G   R   C   L   F   D   V
ccg ggc ttt agg acg ttt gat ggg tca aga acg gta cta tga ccc taa aca atg agg acc
 P   G   F   R   T   F   D   G   S   R   T   V   L   -   P   -   T   M   R   T
aga gat ggt tat aat gag ggc gtt gtc gat act aga aaa ttg caa cca aca tcc tct ctt
 R   D   G   Y   N   E   G   V   V   D   T   R   K   L   Q   P   T   S   S   L
cga aga gtt tat tga att tgt cat caa agg gga taa gta cgg att ggg tac taa act ccc
 R   R   V   Y   -   I   C   H   Q   R   G   -   V   R   I   G   Y   -   T   P
agg ctt ttt gga taa ttt aca ggc aat gta cgc caa agc cag aga tgt gat gcc cga tta
```

Supplementary material S5

```
 R   L   F   G   -   F   T   G   N   V   R   Q   S   Q   R   C   D   A   R   L
tgg tgg ctt cgt taa tga taa tat gaa gcg caa agg aat cct ttc atg gaa ggt agt cca
 W   W   L   R   -   -   -   Y   E   A   Q   R   N   P   F   M   E   G   S   P
ata cct aat taa tcg gta agc gag atg gtg cag taa acc att
 I   P   N   -   S   V   S   E   M   V   Q   -   T   I
```

5'3' Frame 2

```
gtaa att ttc aaa att ttc tat ttc tat gaa att tta aat tgc gtg att tac tga aaa cgc
  -   I   F   K   I   F   Y   F   Y   E   I   L   N   C   V   I   Y   -   K   R
tta cag tgg cag ata act gta tac att tca gga ggt tag ttt ctt gtg att aag aag cta
 L   Q   W   Q   I   T   V   Y   I   S   G   G   -   F   L   V   I   K   K   L
aac ttg aag gac tat ttc atc ctt ccc aat cag ggt tta cag tct tat tta cac aag acg
 N   L   K   D   Y   F   I   L   P   N   Q   G   L   Q   S   Y   L   H   K   T
gag gta ggt agc gac ctt gac att cgt act ccg ttt tgg gac gag aag cct aga tca tcg
 E   V   G   S   D   L   D   I   R   T   P   F   W   D   E   K   P   R   S   S
atc atg gcg atg tga caa gct gtg gtt gac tct tca aat ctg gaa gct aag ttg cca ggt
 I   M   A   M   -   Q   A   V   V   D   S   S   N   L   E   A   K   L   P   G
ttg tat aaa ttt gag atg gac atg aaa tca aaa gtc ggt cca ctc tcc gtc caa gaa ccc
 L   Y   K   F   E   M   D   M   K   S   K   V   G   P   L   S   V   Q   E   P
tta cta aga cgg att ccc gac gta atg gac tat tat aac gat ccg aaa ggt cag gaa gtt
 L   L   R   R   I   P   D   V   M   D   Y   Y   N   D   P   K   G   Q   E   V
agt ccg cga gca atc gca tgc gtt aag gaa gaa ttt aaa gca tta agg ggt ctc agg ttg
 S   P   R   A   I   A   C   V   K   E   E   F   K   A   L   R   G   L   R   L
cgt gat gct gat gca act gtc gct gag atg cgc cgc aat act aat tcg ggc gct cct tat
 R   D   A   D   A   T   V   A   E   M   R   R   N   T   N   S   G   A   P   Y
ttc act cgt cgg tca cgt gtc gag tcg gaa tcc cta gaa ttg gct aag act aga tcg tta
 F   T   R   R   S   R   V   E   S   E   S   L   E   L   A   K   T   R   S   L
gca tat cca gca gtt tta ggg tgg aga gga caa gaa ggt ggt att gat act gat aac gtg
 A   Y   P   A   V   L   G   W   R   G   Q   E   G   G   I   D   T   D   N   V
aaa caa cgc gtt gtt tga atg ttt cct ttc caa acg aac atc gcg gaa tta cag ttc tac
 K   Q   R   V   V   -   M   F   P   F   Q   T   N   I   A   E   L   Q   F   Y
cag ccc atg atc cgg gcg att caa gca cat aaa ctc atc cct gcc tat ttg gat cat aca
 Q   P   M   I   R   A   I   Q   A   H   K   L   I   P   A   Y   L   D   H   T
gca gtg gat gct cgg ata acg aat cta ttt gac acc aaa cct acg ggt gat ctt gta gtt
 A   V   D   A   R   I   T   N   L   F   D   T   K   P   T   G   D   L   V   V
gaa acc gat ttc acc aag ttc gac cag tcg ttc tga aca cca tta caa cat gta gcg cgt
 E   T   D   F   T   K   F   D   Q   S   F   -   T   P   L   Q   H   V   A   R
gct gtg tac gcc gac tga ggt cca caa ctt gat aag tga att aga gaa gta ttc ccg gta
 A   V   Y   A   D   -   G   P   Q   L   D   K   -   I   R   E   V   F   P   V
aaa tat aat att ccc atg gta tta tcg aat aat ttg att tcc acc gga cca cac ggc atg
 K   Y   N   I   P   M   V   L   S   N   N   L   I   S   T   G   P   H   G   M
gcg tcg gga tca ggg ggt act aat gct gat gag tcg att gca cac agg gcg tta cag cat
 A   S   G   S   G   G   T   N   A   D   E   S   I   A   H   R   A   L   Q   H
gaa gct gct tta gcg tcg ggt cag act ctc cac cca tcc tca atg gca tat ggg gac gat
 E   A   A   L   A   S   G   Q   T   L   H   P   S   S   M   A   Y   G   D   D
ggt atc tta acg ttc aag ggt atc acc gtt gat aaa gtg gta gca caa tat act aag cat
 G   I   L   T   F   K   G   I   T   V   D   K   V   V   A   Q   Y   T   K   H
gga gtg acc atg aac gag agt aag cag gga gca agc act acc gag tgc gta ttc cta cgc
 G   V   T   M   N   E   S   K   Q   G   A   S   T   T   E   C   V   F   L   R
cgt tga cac tct atg tcc tat cgt aag aac ggg att atg gta ggt gtt tat tcg acg ttc
 R   -   H   S   M   S   Y   R   K   N   G   I   M   V   G   V   Y   S   T   F
cgg gct tta gga cgt ttg atg ggt caa gaa cgg tac tat gac cct aaa caa tga gga cca
 R   A   L   G   R   L   M   G   Q   E   R   Y   Y   D   P   K   Q   -   G   P
gag atg gtt ata atg agg gcg ttg tcg ata cta gaa aat tgc aac caa cat cct ctc ttc
 E   M   V   I   M   R   A   L   S   I   L   E   N   C   N   Q   H   P   L   F
gaa gag ttt att gaa ttt gtc atc aaa ggg gat aag tac gga ttg ggt act aaa ctc cca
 E   E   F   I   E   F   V   I   K   G   D   K   Y   G   L   G   T   K   L   P
ggc ttt ttg gat aat tta cag gca atg tac gcc aaa gcc aga gat gtg atg ccc gat tat
```

Supplementary material S5

```
  G   F   L   D   N   L   Q   A   M   Y   A   K   A   R   D   V   M   P   D   Y
ggt ggc ttc gtt aat gat aat atg aag cgc aaa gga atc ctt tca tgg aag gta gtc caa
  G   G   F   V   N   D   N   M   K   R   K   G   I   L   S   W   K   V   V   Q
tac cta att aat cgg taa gcg aga tgg tgc agt aaa cca
  Y   L   I   N   R   -   A   R   W   C   S   K   P
```

5'3' Frame 3

```
gtaaa ttt tca aaa ttt tct att tct atg aaa ttt taa att gcg tga ttt act gaa aac gct
    K   F   S   K   F   S   I   S   M   K   F   -   I   A   -   F   T   E   N   A
tac agt ggc aga taa ctg tat aca ttt cag gag gtt agt ttc ttg tga tta aga agc taa
  Y   S   G   R   -   L   Y   T   F   Q   E   V   S   F   L   -   L   R   S   -
act tga agg act att tca tcc ttc cca atc agg gtt tac agt ctt att tac aca aga cgg
  T   -   R   T   I   S   S   F   P   I   R   V   Y   S   L   I   Y   T   R   R
agg tag gta gcg acc ttg aca ttc gta ctc cgt ttt ggg acg aga agc cta gat cat cga
  R   -   V   A   T   L   T   F   V   L   R   F   G   T   R   S   L   D   H   R
tca tgg cga tgt gac aag ctg tgg ttg act ctt caa atc tgg aag cta agt tgc cag gtt
  S   W   R   C   D   K   L   W   L   T   L   Q   I   W   K   L   S   C   Q   V
tgt ata aat ttg aga tgg aca tga aat caa aag tcg gtc cac tct ccg tcc aag aac cct
  C   I   N   L   R   W   T   -   N   Q   K   S   V   H   S   P   S   K   N   P
tac taa gac gga ttc ccg acg taa tgg act att ata acg atc cga aag gtc agg aag tta
  Y   -   D   G   F   P   T   -   W   T   I   I   T   I   R   K   V   R   K   L
gtc cgc gag caa tcg cat gcg tta agg aag aat tta aag cat taa ggg gtc tca ggt tgc
  V   R   E   Q   S   H   A   L   R   K   N   L   K   H   -   G   V   S   G   C
gtg atg ctg atg caa ctg tcg ctg aga tgc gcc gca ata cta att cgg gcg ctc ctt att
  V   M   L   M   Q   L   S   L   R   C   A   A   I   L   I   R   A   L   L   I
tca ctc gtc ggt cac gtg tcg agt cgg aat ccc tag aat tgg cta aga cta gat cgt tag
  S   L   V   G   H   V   S   S   R   N   P   -   N   W   L   R   L   D   R   -
cat atc cag cag ttt tag ggt gga gag gac aag aag gtg gta ttg ata ctg ata acg tga
  H   I   Q   Q   F   -   G   G   E   D   K   K   V   V   L   I   L   I   T   -
aac aac gcg ttg ttt gaa tgt ttc ctt tcc aaa cga aca tcg cgg aat tac agt tct acc
  N   N   A   L   F   E   C   F   L   S   K   R   T   S   R   N   Y   S   S   T
agc cca tga tcc ggg cga ttc aag cac ata aac tca tcc ctg cct att tgg atc ata cag
  S   P   -   S   G   R   F   K   H   I   N   S   S   L   P   I   W   I   I   Q
cag tgg atg ctc gga taa cga atc tat ttg aca cca aac cta cgg gtg atc ttg tag ttg
  Q   W   M   L   G   -   R   I   Y   L   T   P   N   L   R   V   I   L   -   L
aaa ccg att tca cca agt tcg acc agt cgt tct gaa cac cat tac aac atg tag cgc gtg
  K   P   I   S   P   S   S   T   S   R   S   E   H   H   Y   N   M   -   R   V
ctg tgt acg ccg act gag gtc cac aac ttg ata agt gaa tta gag aag tat tcc cgg taa
  L   C   T   P   T   E   V   H   N   L   I   S   E   L   E   K   Y   S   R   -
aat ata ata ttc cca tgg tat tat cga ata att tga ttt cca ccg gac cac acg gca tgg
  N   I   I   F   P   W   Y   Y   R   I   I   -   F   P   P   D   H   T   A   W
cgt cgg gat cag ggg gta cta atg ctg atg agt cga ttg cac aca ggg cgt tac agc atg
  R   R   D   Q   G   V   L   M   L   M   S   R   L   H   T   G   R   Y   S   M
aag ctg ctt tag cgt cgg gtc aga ctc tcc acc cat cct caa tgg cat atg ggg acg atg
  K   L   L   -   R   R   V   R   L   S   T   H   P   Q   W   H   M   G   T   M
gta tct taa cgt tca agg gta tca ccg ttg ata aag tgg tag cac aat ata cta agc atg
  V   S   -   R   S   R   V   S   P   L   I   K   W   -   H   N   I   L   S   M
gag tga cca tga acg aga gta agc agg gag caa gca cta ccg agt gcg tat tcc tac gcc
  E   -   P   -   T   R   V   S   R   E   Q   A   L   P   S   A   Y   S   Y   A
gtt gac act cta tgt cct atc gta aga acg gga tta tgg tag gtg ttt att cga cgt tcc
  V   D   T   L   C   P   I   V   R   T   G   L   W   -   V   F   I   R   R   S
ggg ctt tag gac gtt tga tgg gtc aag aac ggt act atg acc cta aac aat gag gac cag
  G   L   -   D   V   -   W   V   K   N   G   T   M   T   L   N   N   E   D   Q
aga tgg tta taa tga ggg cgt tgt cga tac tag aaa att gca acc aac atc ctc tct tcg
  R   W   L   -   -   G   R   C   R   Y   -   K   I   A   T   N   I   L   S   S
```

Supplementary material S5

```
aag agt tta ttg aat ttg tca tca aag ggg ata agt acg gat tgg gta cta aac tcc cag
 K   S   L   L   N   L   S   S   K   G   I   S   T   D   W   V   L   N   S   Q
gct ttt tgg ata att tac agg caa tgt acg cca aag cca gag atg tga tgc ccg att atg
 A   F   W   I   I   Y   R   Q   C   T   P   K   P   E   M   -   C   P   I   M
gtg gct tcg tta atg ata ata tga agc gca aag gaa tcc ttt cat gga agg tag tcc aat
 V   A   S   L   M   I   I   -   S   A   K   E   S   F   H   G   R   -   S   N
acc taa tta atc ggt aag cga gat ggt gca gta aac cat
 T   -   L   I   G   K   R   D   G   A   V   N   H
```
