# Supplementary material for: Detection and Molecular Characterization of Picobirnaviruses (PBVs) in the Mongoose: Identification of a Novel PBV Using an Alternative Genetic Code
Source: Viruses. 2020 Jan 15;12(1):99. doi: 10.3390/v12010099 (PMC7019992; doi:10.3390/v12010099)
Supplement: Supplementary file 1 [file viruses-12-00099-s001.zip › Supplementary material S6.pdf]

Supplementary material S6. Translation (5'-3', frame 2) of the novel mongoose PBV-like sequence M17A using an alternative mold mitochondrial genetic code. The translation frame was generated by the ORF finder (https://www.ncbi.nlm.nih.gov/orffinder/, accessed 24rd November 2019). The putative RNA-dependent RNA polymerase and corresponding coding sequence are shown with pink.

```
gtaa att ttc aaa att ttc tat ttc tat gaa att tta aat tgc gtg att tac tga aaa cgc
  -   I   F   K   I   F   Y   F   Y   E   I   L   N   C   V   I   Y   W   K   R
tta cag tgg cag ata act gta tac att tca gga ggt tag ttt ctt gtg att aag aag cta
 L   Q   W   Q   I   T   V   Y   I   S   G   G   -   F   L   M   I   K   K   L
aac ttg aag gac tat ttc atc ctt ccc aat cag ggt tta cag tct tat tta cac aag acg
 N   L   K   D   Y   F   I   L   P   N   Q   G   L   Q   S   Y   L   H   K   T
gag gta ggt agc gac ctt gac att cgt act ccg ttt tgg gac gag aag cct aga tca tcg
 E   V   G   S   D   L   D   I   R   T   P   F   W   D   E   K   P   R   S   S
atc atg gcg atg tga caa gct gtg gtt gac tct tca aat ctg gaa gct aag ttg cca ggt
 I   M   A   M   W   Q   A   V   V   D   S   S   N   L   E   A   K   L   P   G
ttg tat aaa ttt gag atg gac atg aaa tca aaa gtc ggt cca ctc tcc gtc caa gaa ccc
 L   Y   K   F   E   M   D   M   K   S   K   V   G   P   L   S   V   Q   E   P
tta cta aga cgg att ccc gac gta atg gac tat tat aac gat ccg aaa ggt cag gaa gtt
 L   L   R   R   I   P   D   V   M   D   Y   Y   N   D   P   K   G   Q   E   V
agt ccg cga gca atc gca tgc gtt aag gaa gaa ttt aaa gca tta agg ggt ctc agg ttg
 S   P   R   A   I   A   C   V   K   E   E   F   K   A   L   R   G   L   R   L
cgt gat gct gat gca act gtc gct gag atg cgc cgc aat act aat tcg ggc gct cct tat
 R   D   A   D   A   T   V   A   E   M   R   R   N   T   N   S   G   A   P   Y
ttc act cgt cgg tca cgt gtc gag tcg gaa tcc cta gaa ttg gct aag act aga tcg tta
 F   T   R   R   S   R   V   E   S   E   S   L   E   L   A   K   T   R   S   L
gca tat cca gca gtt tta ggg tgg aga gga caa gaa ggt ggt att gat act gat aac gtg
 A   Y   P   A   V   L   G   W   R   G   Q   E   G   G   I   D   T   D   N   V
aaa caa cgc gtt gtt tga atg ttt cct ttc caa acg aac atc gcg gaa tta cag ttc tac
 K   Q   R   V   V   W   M   F   P   F   Q   T   N   I   A   E   L   Q   F   Y
cag ccc atg atc cgg gcg att caa gca cat aaa ctc atc cct gcc tat ttg gat cat aca
 Q   P   M   I   R   A   I   Q   A   H   K   L   I   P   A   Y   L   D   H   T
gca gtg gat gct cgg ata acg aat cta ttt gac acc aaa cct acg ggt gat ctt gta gtt
 A   V   D   A   R   I   T   N   L   F   D   T   K   P   T   G   D   L   V   V
gaa acc gat ttc acc aag ttc gac cag tcg ttc tga aca cca tta caa cat gta gcg cgt
 E   T   D   F   T   K   F   D   Q   S   F   W   T   P   L   Q   H   V   A   R
gct gtg tac gcc gac tga ggt cca caa ctt gat aag tga att aga gaa gta ttc ccg gta
 A   V   Y   A   D   W   G   P   Q   L   D   K   W   I   R   E   V   F   P   V
aaa tat aat att ccc atg gta tta tcg aat aat ttg att tcc acc gga cca cac ggc atg
 K   Y   N   I   P   M   V   L   S   N   N   L   I   S   T   G   P   H   G   M
gcg tcg gga tca ggg ggt act aat gct gat gag tcg att gca cac agg gcg tta cag cat
 A   S   G   S   G   G   T   N   A   D   E   S   I   A   H   R   A   L   Q   H
gaa gct gct tta gcg tcg ggt cag act ctc cac cca tcc tca atg gca tat ggg gac gat
 E   A   A   L   A   S   G   Q   T   L   H   P   S   S   M   A   Y   G   D   D
ggt atc tta acg ttc aag ggt atc acc gtt gat aaa gtg gta gca caa tat act aag cat
 G   I   L   T   F   K   G   I   T   V   D   K   V   V   A   Q   Y   T   K   H
gga gtg acc atg aac gag agt aag cag gga gca agc act acc gag tgc gta ttc cta cgc
 G   V   T   M   N   E   S   K   Q   G   A   S   T   T   E   C   V   F   L   R
cgt tga cac tct atg tcc tat cgt aag aac ggg att atg gta ggt gtt tat tcg acg ttc
 R   W   H   S   M   S   Y   R   K   N   G   I   M   V   G   V   Y   S   T   F
cgg gct tta gga cgt ttg atg ggt caa gaa cgg tac tat gac cct aaa caa tga gga cca
 R   A   L   G   R   L   M   G   Q   E   R   Y   Y   D   P   K   Q   W   G   P
gag atg gtt ata atg agg gcg ttg tcg ata cta gaa aat tgc aac caa cat cct ctc ttc
 E   M   V   I   M   R   A   L   S   I   L   E   N   C   N   Q   H   P   L   F
```

gaa gag ttt att gaa ttt gtc atc aaa ggg gat aag tac gga ttg ggt act aaa ctc cca
E E F I E F V I K G D K Y G L G T K L P
ggc ttt ttg gat aat tta cag gca atg tac gcc aaa gcc aga gat gtg atg ccc gat tat
G F L D N L Q A M Y A K A R D V M P D Y
ggt ggc ttc gtt aat gat aat atg aag cgc aaa gga atc ctt tca tgg aag gta gtc caa
G G F V N D N M K R K G I L S W K V V Q
tac cta att aat cgg taa gcg aga tgg tgc agt aaa cca
Y L I N R - A R W C S K P
