# Supplementary material for: Detection and Molecular Characterization of Picobirnaviruses (PBVs) in the Mongoose: Identification of a Novel PBV Using an Alternative Genetic Code
Source: Viruses. 2020 Jan 15;12(1):99. doi: 10.3390/v12010099 (PMC7019992; doi:10.3390/v12010099)
Supplement: Supplementary file 1 [file viruses-12-00099-s001.zip › Supplementary material S7.pdf]

Supplementary material S7. Translation (5'-3', frame 2) of the novel mongoose PBV-like sequence M17A using an alternative invertebrate mitochondrial genetic code. The translation frame was generated by the ORF finder (https://www.ncbi.nlm.nih.gov/orffinder/, accessed 23rd November 2019). The putative RNA-dependent RNA polymerase and corresponding coding sequence are shown with pink.

gtaa att ttc aaa att ttc tat ttc tat gaa att tta aat tgc gtg att tac tga aaa cgc

- I F K I F Y F Y E I L N C V I Y W K R

tta cag tgg cag ata act gta tac att tca gga ggt tag ttt ctt gtg att aag aag cta

L Q W Q M T V Y I S G G - F L M I K K L

aac ttg aag gac tat ttc atc ctt ccc aat cag ggt tta cag tct tat tta cac aag acg

N L K D Y F I L P N Q G L Q S Y L H K T

gag gta ggt agc gac ctt gac att cgt act ccg ttt tgg gac gag aag cct aga tca tcg

E V G S D L D I R T P F W D E K P S S S

atc atg gcg atg tga caa gct gtg gtt gac tct tca aat ctg gaa gct aag ttg cca ggt

I M A M W Q A V V D S S N L E A K L P G

ttg tat aaa ttt gag atg gac atg aaa tca aaa gtc ggt cca ctc tcc gtc caa gaa ccc

L Y K F E M D M K S K V G P L S V Q E P

tta cta aga cgg att ccc gac gta atg gac tat tat aac gat ccg aaa ggt cag gaa gtt

L L S R I P D V M D Y Y N D P K G Q E V

agt ccg cga gca atc gca tgc gtt aag gaa gaa ttt aaa gca tta agg ggt ctc agg ttg

S P R A I A C V K E E F K A L S G L S L

cgt gat gct gat gca act gtc gct gag atg cgc cgc aat act aat tcg ggc gct cct tat

R D A D A T V A E M R R N T N S G A P Y

ttc act cgt cgg tca cgt gtc gag tcg gaa tcc cta gaa ttg gct aag act aga tcg tta

F T R R S R V E S E S L E L A K T S S L

gca tat cca gca gtt tta ggg tgg aga gga caa gaa ggt ggt att gat act gat aac gtg

A Y P A V L G W S G Q E G G I D T D N V

aaa caa cgc gtt gtt tga atg ttt cct ttc caa acg aac atc gcg gaa tta cag ttc tac

K Q R V V W M F P F Q T N I A E L Q F Y

cag ccc atg atc cgg gcg att caa gca cat aaa ctc atc cct gcc tat ttg gat cat aca

Q P M I R A I Q A H K L I P A Y L D H T

gca gtg gat gct cgg ata acg aat cta ttt gac acc aaa cct acg ggt gat ctt gta gtt

A V D A R M T N L F D T K P T G D L V V

gaa acc gat ttc acc aag ttc gac cag tcg ttc tga aca cca tta caa cat gta gcg cgt

E T D F T K F D Q S F W T P L Q H V A R

gct gtg tac gcc gac tga ggt cca caa ctt gat aag tga att aga gaa gta ttc ccg gta

A V Y A D W G P Q L D K W I S E V F P V

aaa tat aat att ccc atg gta tta tcg aat aat ttg att tcc acc gga cca cac ggc atg

K Y N I P M V L S N N L I S T G P H G M

gcg tcg gga tca ggg ggt act aat gct gat gag tcg att gca cac agg gcg tta cag cat

A S G S G G T N A D E S I A H S A L Q H

gaa gct gct tta gcg tcg ggt cag act ctc cac cca tcc tca atg gca tat ggg gac gat

E A A L A S G Q T L H P S S M A Y G D D

ggt atc tta acg ttc aag ggt atc acc gtt gat aaa gtg gta gca caa tat act aag cat

G I L T F K G I T V D K V V A Q Y T K H

gga gtg acc atg aac gag agt aag cag gga gca agc act acc gag tgc gta ttc cta cgc

G V T M N E S K Q G A S T T E C V F L R

cgt tga cac tct atg tcc tat cgt aag aac ggg att atg gta ggt gtt tat tcg acg ttc

R W H S M S Y R K N G I M V G V Y S T F

cgg gct tta gga cgt ttg atg ggt caa gaa cgg tac tat gac cct aaa caa tga gga cca

R A L G R L M G Q E R Y Y D P K Q W G P

gag atg gtt ata atg agg gcg ttg tcg ata cta gaa aat tgc aac caa cat cct ctc ttc

E M V M M S A L S M L E N C N Q H P L F

gaa gag ttt att gaa ttt gtc atc aaa ggg gat aag tac gga ttg ggt act aaa ctc cca
 E   E   F   I   E   F   V   I   K   G   D   K   Y   G   L   G   T   K   L   P
ggc ttt ttg gat aat tta cag gca atg tac gcc aaa gcc aga gat gtg atg ccc gat tat
 G   F   L   D   N   L   Q   A   M   Y   A   K   A   S   D   V   M   P   D   Y
ggt ggc ttc gtt aat gat aat atg aag cgc aaa gga atc ctt tca tgg aag gta gtc caa
 G   G   F   V   N   D   N   M   K   R   K   G   I   L   S   W   K   V   V   Q
tac cta att aat cgg taa gcg aga tgg tgc agt aaa cca
 Y   L   I   N   R   -   A   S   W   C   S   K   P
