# Supplementary material for: Detection and Molecular Characterization of Picobirnaviruses (PBVs) in the Mongoose: Identification of a Novel PBV Using an Alternative Genetic Code
Source: Viruses. 2020 Jan 15;12(1):99. doi: 10.3390/v12010099 (PMC7019992; doi:10.3390/v12010099)
Supplement: Supplementary file 1 [file viruses-12-00099-s001.zip › Supplementary material S8.pdf]

Supplementary material S8. Alignment of the putative RNA-dependent RNA polymerase (RdRp) of the novel mongoose PBV-like strain M17A with those of the prototype human genogroup-I PBV strain (PBV/Human/CHN/1-CHN-97/1997) and mongoose PBV GI strain PBV/Mongoose/KNA/M17B/2017. The M17A and M17B RdRp sequences were identified in the same fecal sample. The putative M17A RdRp translated using the mold mitochondrial genetic code and the invertebrate mitochondrial genetic code is shown with red and green font, respectively. Amino acid (aa) mismatches between putative RdRps generated using the alternative mitochondrial genetic codes (that of mold and invertebrate) are highlighted with blue. The three conserved domains in the putative RdRps are highlighted with yellow. A '*' denotes an identical aa residue, whilst '-' indicates absence of an aa residue. Numbers to the right indicate the positions of the aa for respective PBV strains.

```
PBV/Human/CHN/1-CHN-97/1997        -MPKNNEVSFG----------EYFNLP-NPGLKSYFDRVRSGNTDEYRTT 38
PBV-like/Mongoose/M17A/2017        MIKKLNLK-------------DYFILP-NQGLQSYLHKTEVGSDLDIRTP 36
PBV-like/Mongoose/M17A/2017        MIKKLNLK-------------DYFILP-NQGLQSYLHKTEVGSDLDIRTP 36
PBV/Mongoose/M17B/2017             -MPKNNVTSFINNHFVDTNYTSYFTHSGFQSLRTYMGNVVKGQPKVYTAP 49
                                      * *                **       *  *      *

PBV/Human/CHN/1-CHN-97/1997        FFRGRSLDSILKAWKPTLDK--IENDWPTLLDFENDLAKKVGPLSIQKPL 86
PBV-like/Mongoose/M17A/2017        FWDEKPRSSIMAMWQAVVDSSNLEAKLPGLYKFEMDMKSKVGPLSVQEPL 86
PBV-like/Mongoose/M17A/2017        FWDEKPSSSIMAMWQAVVDSSNLEAKLPGLYKFEMDMKSKVGPLSVQEPL 86
PBV/Mongoose/M17B/2017             FAKGSTIQSVLDDWQEVLVS--ISDKWPSLWEFEMDLASKVGPLSVQKPL 97
                                   *       *    *             * *  ** *   ****** * **

PBV/Human/CHN/1-CHN-97/1997        AERLDDIESYYESISLDSEPISRSAIAAVIAEWGDARGIQLRGQRKTVDN 136
PBV-like/Mongoose/M17A/2017        LRRIPDVMDYYNDP--KGQEVSPRAIACVKEEFKALRGLRLRDADATVAE 134
PBV-like/Mongoose/M17A/2017        LSRIPDVMDYYNDP--KGQEVSPRAIACVKEEFKALSGLSLRDADATVAE 134
PBV/Mongoose/M17B/2017             RERITDIAHYYEDILLSSTPIDPVAIKQTIGEFNKARGLRRRSQARTVEM 147
                                     *  *   **             **     *     *   *    **

PBV/Human/CHN/1-CHN-97/1997        MKKSTNSGSPFFTKRRAVVDKTIPARLDY-------LGVQTKQFLNNSDW 179
PBV-like/Mongoose/M17A/2017        MRRNTNSGAPYFTRRSRVESESL-------------------ELAKTRSL 165
PBV-like/Mongoose/M17A/2017        MRRNTNSGAPYFTRRSRVESESL-------------------ELAKTSSL 165
PBV/Mongoose/M17B/2017             MKKSTNSGNPFFTKRRNVVLDTFPCGVDYTPNPVYYLQDAYQMLPNNNDF 197
                                   *   **** * ** *  *

PBV/Human/CHN/1-CHN-97/1997        YSAAVLGWRGQEGGPTDEDVKQRVVWMFPFGVNVKELQFYQPAIEIAQKR 229
PBV-like/Mongoose/M17A/2017        AYPAVLGWRGQEGGIDTDNVKQRVVWMFPFQTNIAELQFYQPMIRAIQAH 215
PBV-like/Mongoose/M17A/2017        AYPAVLGWSGQEGGIDTDNVKQRVVWMFPFQTNIAELQFYQPMIRAIQAH 215
PBV/Mongoose/M17B/2017             KACAVIGWRGQEGGPEDEDVKQRVVWMFPFAVNIEELTVYQPLIETFQRF 247
                                      ** ** *****     ***********  *  **  *** *   *

PBV/Human/CHN/1-CHN-97/1997        WITPAWIGMEAVDQRITKLFDTKAKSELVICTDFSKFDQHFNKDMQDAAR 279
PBV-like/Mongoose/M17A/2017        KLIPAYLDHTAVDARITNLFDTKPTGDLVVETDFTKFDQSFWTPLQHVAR 265
PBV-like/Mongoose/M17A/2017        KLIPAYLDHTAVDARMTNLFDTKPTGDLVVETDFTKFDQSFWTPLQHVAR 265
PBV/Mongoose/M17B/2017             NLVPAWVSMESVDMEVTRLFDTKNSDDLVVCTDFTKFDQHFNVDLQNAAK 297
                                      **      **   * *****    **  *** **** *    *  *

PBV/Human/CHN/1-CHN-97/1997        TIIAALLNGSSESRDWLDNVFPVKYAIPLAMDMG----VVKTGFHGMGSG 325
PBV-like/Mongoose/M17A/2017        AVYADWG---PQLDKWIREVFPVKYNIPMVLSNN----LISTGPHGMASG 308
PBV-like/Mongoose/M17A/2017        AVYADWG---PQLDKWISEVFPVKYNIPMVLSNN----LISTGPHGMASG 308
PBV/Mongoose/M17B/2017             TILQWIMSRERNVDDWFENVFGIKYMIPLLWEAQADIVSVFHGFHGMASG 347
                                                  *   **  ** **   .          * *** **

PBV/Human/CHN/1-CHN-97/1997        SGGTNFDETLTHRALQYEAAQKAKSVLNPNSQCLGDDGILSYPGITVEDV 375
PBV-like/Mongoose/M17A/2017        SGGTNADESIAHRALQHEAALASGQTLHPSSMAYGDDGILTFKGITVDKV 358
PBV-like/Mongoose/M17A/2017        SGGTNADESIAHSALQHEAALASGQTLHPSSMAYGDDGILTFKGITVDKV 358
PBV/Mongoose/M17B/2017             SGGTNVDETLAHRALQREAAIKSKQILNPHSMCLGDDGILSYPGITVNEV 397
                                   ***** **   * *** ***      * * *   ******   ****  *

PBV/Human/CHN/1-CHN-97/1997        TRSYSAHGQEMNIDKQYASAHDCTYLRRWHHDDYRVDGVCVGVYATYRAL 425
PBV-like/Mongoose/M17A/2017        VAQYTKHGVTMNESKQGASTTECVFLRRWHSMSYRKNGIMVGVYSTFRAL 408
PBV-like/Mongoose/M17A/2017        VAQYTKHGVTMNESKQGASTTECVFLRRWHSMSYRKNGIMVGVYSTFRAL 408
PBV/Mongoose/M17B/2017             VDAYQSHGQECNLSKQYASTQDCVYLRRWHHKDYRDNGVCVGVYSTCRAL 447
                                      *  **   *  ** **   *  *****   **  *  **** * ***

PBV/Human/CHN/1-CHN-97/1997        GRLCEQERYYDPEVWGPKMVALRQLSIIENCKYHPLREEFVNFCMKGDKY 475
PBV-like/Mongoose/M17A/2017        GRLMGQERYYDPKQWGPEMVIMRALSILENCNQHPLFEEFIEFVIKGDKY 458
PBV-like/Mongoose/M17A/2017        GRLMGQERYYDPKQWGPEMVMMSALSMLENCNQHPLFEEFIEFVIKGDKY 458
PBV/Mongoose/M17B/2017             GRLRYLERFMDPEYWSPQMVALRQLSIIENCKYHPLRDEFAEFCMKRDKY 497
                                   ***   **  **  * * **    **  ***  ***  **  *  * ***

PBV/Human/CHN/1-CHN-97/1997        RLGLDIPGFLDNIEKLAMEATDLMPDFLGYTKSLGTSGDRGIGQWWVVNY 525
PBV-like/Mongoose/M17A/2017        GLGTKLPGFLDNLQAMYAKARDVMPDYGGFVN--DNMKRKGILSWKVVQY 506
PBV-like/Mongoose/M17A/2017        GLGTKLPGFLDNLQAMYAKASDVMPDYGGFVN--DNMKRKGILSWKVVQY 506
PBV/Mongoose/M17B/2017             RLGIDIPGFLDKIATYAKESIDHMPDFLGYTKTLQGFKPTGISEWWIVKY 547
                                    **   *****          * ***  *           **  *  * *

PBV/Human/CHN/1-CHN-97/1997        LKSLR 530
PBV-like/Mongoose/M17A/2017        LINR- 510
PBV-like/Mongoose/M17A/2017        LINR- 510
PBV/Mongoose/M17B/2017             LKSHR 552
                                   *
```
